# The fixed idea of sex and the dawn of theoretical gender medicine

**DOI:** 10.1017/mdh.2025.10017

**Published:** 2025-07

**Authors:** Diederik Janssen

**Affiliations:** Maastricht University, Graduate School of Arts and Social Sciences, Maastricht, The Netherlands

**Keywords:** gender identity, Richard Freiherr von Krafft-Ebing (1840-1902), Théodule-Armand Ribot (1839–1916), transgender, Rudolph Arndt (1835–1900), monomania

## Abstract

Cross-gender behaviour gradually entered the spheres of aetiology and diagnosis during the eighteenth century with reference to scattered instances of male cross-dressing. But well into the nineteenth century, “gender identity” (a mid-twentieth-century term) remained a poorly theorised instance of medicalisation. Late eighteenth-century concepts of “dynamic hermaphroditism” accounted for gender-nonconforming behaviours and aspirations, but could not account for the observed heterogeneity in disparities between sexed body and mind. Increasingly substantive contributions to aetiology were seen during the late 1870s and 1880s, particularly in response to Carl Westphal’s convoluted, 1869 concept of “contrary sexual feeling” (*conträre Sexualempfindung*). Richard von Krafft-Ebing’s notion of *metamorphosis sexualis paranoica* provided one of the most authoritative approaches to the question of gender identification in “sexual inversion”. The notion, which took the first seven German editions of his *Psychopathis sexualis* to achieve a definitive formulation, needs to be seen in light of Krafft-Ebing’s earlier conceptions of sexual delusion, which straddled the realms of the experienced sexual body and sense of self. Moreover, Krafft-Ebing was not the first to outline a theory of non-cisgender identity, as demonstrated by the mid-1880s work of Théodule-Armand Ribot and Rudolph Arndt, as well as various significantly earlier approaches to what had been considered the “monomania of sexual transformation”.

Pinpointing gender medicine’s birthdate is challenging. While *gender* is a 1940s/1950s medical-psychological Americanism, some eighteenth- and various nineteenth-century European texts are quite reminiscent of its present-day clinical challenges and politicisations. Consider one by Joseph Jörger (1860–1933), physician at the St. Pirminsberg psychiatric clinic in Pfäfers (Switzerland), detailing a complex case of the presumptive ‘delusional masculinity’ (*wahnhafte Männlichkeit*) or ‘sex/gender delusions’ (*Geschlechtswahne*) of natural philosophy student Agnes/Arnold, aged 25. For several years, Agnes had been ‘aware of his real sex [*seines wirklichen Geschlechts*] and now also wears men’s clothes’, spoke with a low voice, and exclusively fancied women.^1^ His mother, described as notoriously and long-term ‘paranoid’, sought counsel with a physician to confirm what would have been an error in sex assignment. A physician had found no anatomical abnormalities, proposing a diagnosis of ‘chronic mental disorder and a so-called perverse sexual feeling as the basis of this delusion’. Jörger dissented, however, judging from a life story written down at the St. Pirminsberg as well as extensive clinical records on the patient, mother, and sister. Arnold suggested that both of his parents had raised him as a boy, he subsequently discovered ‘homosexual’ feelings, and only later ‘became aware of my masculinity’ (‘*Meine Männlichkeit kam mir erst … recht zum Bewusstsein*’), which he tried to make sense of through Darwin after a lecture on related matters attended while in the U.S., and ended up construing in terms of male intersexuality (‘hermaphroditism’). The mother came to be fully convinced of the verdict, as did an apparently unlicensed American doctor, though the latter never performed a physical examination. The mother, however, went on to claim that Arnold impregnated his sister. During their stay in the U.S., the mother, who had advised a physician of her ‘daughter’s homosexuality’ was advised by both the physician and the police, to have him dress as a male. Suicidal, Arnold requested a physician to send him to an insane asylum, barring encounters with women. Yet upon discovery of his female body, he was sent from the men’s to the women’s ward. Some longstanding body image problems would have been catalysts, it was thought: self-consciousness about Arnold’s appearance worsened while a university student, and Arnold’s voice would have ‘broken’ in his early twenties. Weighing interpretative options, Jörger ultimately concluded that ‘This new delusional system of Ag[nes] seems to me the product of her masculine upbringing and her distress, and in no way the result of a perverse sexual disposition’.^2^

Although much more could be said about this early vignette of gender dysphoria consultation *avant la lettre*, several aspects seem noteworthy: the awareness-of-one’s-sex being teased apart from questions of sexual orientation, though clearly still uninformed by a concept of gender identity, hence understood as delusional; the insinuation of novel, *récherché* (Darwinian) frames into patients’ attempts at self-understanding; nosological frames of what the French called *folie imposée* and *folie communiquée* being projected around the entire familial system as it, for years, had been trying to make sense of ‘sex’ in their now young adult son; finally, various forms of *savoir* and distress concerning sexed selfhood ultimately being traced back to and adjudicated on the basis of their seeming origins, leading to an indictment of – in this case affirmative – upbringing. These elements seem hardly alien to the often-bitter polemics concerning gender dysphoria in present-day popular debate in the United States. A defining and heavily politicised point of divergence is still the role of the social environment in gender identity development, today prominently including the role of the conceptual apparatus – the very notion – of gender identity itself.

As has been well-researched, the work of this apparatus has been intricate and shifting, historically suspending what are today understood as non-cisgender identities between psychiatrisation, depsychiatrisation, and imminent repsychiatrisation. While longstanding notions of ‘gender identity disorder’ (DSM-III through IV-TR) were abandoned by the 2013 DSM-5 for *gender dysphoria*, U.S. populist right-wing rhetoric has increasingly construed adult transgender identification as a ‘delusional’ disorder of sorts and the rising visibility of trans persons on the public stage, as well as rising numbers of referrals, as evidencing ‘mass delusion’ relying on ‘propaganda’ and ‘indoctrination.’ From 2017, less straightforwardly political approaches have posited that nontrivial ‘social contagion’ effects attributed to the rising cultural prominence of questions of gender would indeed explain some of the evidently changeable presentation, especially since the publication of the DSM-5, of adolescent gender dysphoria. These often-divisive disputations animate the politically loaded problem of teasing apart the historical *idea* of gender identity and the many expressions and manifestations of identity and dysphoria that idea, historically, has aimed to name, classify, and treat. Gender identity/dysphoria has thus emerged as one of several hyphenated sites – sexual orientation, sexual phobia, sexual trauma – where a historically medical apparatus of scientific pronouncements has come to define an increasingly discursive modern Western experience of selfhood. But if anything, Jörger’s case suggests that this happened well in advance of ‘gender identity’ and indeed of Magnus Hirschfeld’s well-known overhaul of nineteenth-century nosology in terms of ‘*Transvestitismus*’.

Below I take seriously these allusions to a medical prehistory of Hirschfeld’s ‘transvestites’, noting that to date this question has been too narrowly informed by the angles of intersexuality and sexual orientation. I propose broadening these two angles by looking at collateral medical-conceptual attention to the problem of ‘sex’ as a *fixed idea* and a self-attributed dimension of selfhood. I observe that aetiological interest in the already ageing delusional (fixed idea) models of misrecognized ‘sex-of-self’ culminated in the 1880s, then tentatively moving toward what I argue were more general developmental-psychological understandings of ‘sex’ as a self-attributed identity aspect. Below, I discuss (1) this nosological frame for sex as *idée fixe*, (2) the growing aetiological aspiration it came to secure, and finally (3) three 1880s aetiologists who, in the process, began to wonder about normal sex-of-self (‘gender’) development. Of necessity, I will be paying attention to how such a dimension of selfhood was discussed before it was called an *Identität/identité*, using ‘sex’ within scare quotes to translate European terms (*Geschlecht, sexe*) then applied to what were quite heterogenous instances of self-identification. Late nineteenth-century anticipations of gender identity can be seen transpiring apropos multiple aetiological approaches to a heterogeneous archive of clinical presentations where the sex-of-self appeared in doubt or inconstant.

## The fixed idea of sex

Intersex conditions and non-heterosexuality are two well-established sites for encountering early medical circumscriptions of one’s identification as male or female. The European late nineteenth century is often nominated as a period of ‘emergence’ or ‘invention’ in these two respects. According to Geertje Mak and Rainer Herrn, among others, it is only after the turn of the nineteenth century that ‘hermaphroditism’ (intersex) cases came to illustrate a ‘transformation from sex as inscribed in a person to sex as ingrained in the self’.^3^ Scholarship on this frame shift, from sex attached to the legal entity of a person to an experienced, expressed *sex of self* (Herrn speaks of *subjektive Geschlechtszugehörigkeit*), elaborated on earlier scholarship on the sexual self of sodomites and tribades in the eighteenth and early nineteenth centuries, categories cross-fading with collectivizing lesbian and gay identities – ‘a dramatic narration with the true, inner sexual self as outcome.’^4^ This would have paved the way to a ‘new autobiographical script available for the life history of a person with a so-called “error of sex”’, a script echoing the older metropolitan ones of the gay male: the *molly*, *urning*, *warme Bruder.* ^5^ The new script and attention *turned sex inward*, noted Mak: ‘what had previously been indicators for a person’s sexed position in society, now pointed to lifelong inclinations, preferences and aversions which characterized the sex of a person’s inner, genuine being.’^6^ Mak more recently maintained that it was ‘only through medical cases of (pseudo-)hermaphroditism and psychiatric cases of ‘sexual inversion’ that the idea of an inner, inalienable psychological ‘sex of self’ started to take shape’.^7^ The mid-twentieth-century nominal introduction of ‘gender identity’ in American medical psychology has been understood as the outcome of clinical engagements with similar problems concerning sexual differentiation, including adrenal hyperplasia. Gender identity, Jules Gill-Peterson proposed, was ‘the endpoint of a teleology of growth out of plasticity […] a psychosocial dimension of sex, rather than a separable ontological entity’.^8^

What has remained undiscussed in this scholarship are considerably older frames for ‘misidentification of sex’ as a space of alienation, namely those of melancholic delusion and monomania. Proto-psychiatric nosological slots for ‘melancholic’ misidentification of one’s sex were advanced as early as the early 1730s. The Scythian, ‘feminine malady’ (*θήλεα νοῦσος*) of both Herodotean and Hippocratic fame, was one of multiple sites where this interpretative innovation took place.^9^ By the 1730s, legal and nosological considerations in France independently spawned explicitly psychiatricising frames for Scythian but also modern gender-crossing, rendering it a distinct form of *melancholia* or *vapours.*

Several vignettes and *causes célèbres* animated this modern aetiological turn. Ubiquitous eighteenth-century characterisations of male cross-dressing in popular terms of *manie* or *démence* followed the 1737 gossipy and perhaps fabricated *Mémoires* of cross-dressing abbot François Timoléon, abbé de Choisy (1644–1724), for instance. De Choisy made for one of the various cases of cross-dressing ‘mania.’ As in Jörger’s case chronicled above, scrutiny extended to Choisy’s mother, who reportedly cross-dressed them as a child, indeed on the basis of what would be a singular mania. Build-up to these qualifications is captured by an opinion provided by polymath Jean le Rond d’Alembert (1717–1783): ‘The kind of taste that he [Choisy] retained too long for such a strange and blameworthy travesty [*travestissement*], is sad proof of the unfortunate empire that the first nonsense, with which a bad education has infected them, retains over certain minds.’^10^ More specifically: ‘This species of dementia [*démence*] (for why not call it by its name?) would […] have been only madness without consequence, if the Abbé de Choisy had not abused it in a very serious circumstance’.^11^ Childhood habituation was indeed an aetiological reading widely ventilated as soon as De Choisy’s biography came out.^12^ Widespread nineteenth-century attention to similar instances routinely referenced eighteenth-century exemplars such as this one, references animated by various quasi-medical verdicts – *manie efféminée*, *manie de se déguiser en femme*, *manie de s’habiller comme une femme*, *manie de l’habiller en fille.*

It is during the first half of the eighteenth century, then, that an ideational, namely delusional, frame for ‘one’s sex’ came into focus, inviting early speculations concerning aetiology, eventually also early anti-psychiatric arguments. Illustrative is a nineteen-page apologia written in 1831 by French Navy commander Christophe-Paulin de La Poix de Fréminville (1787–1848) concerning his interest in cross-dressing: ‘the inclination that some men have to dress as women […] the passion that leads them to assimilate, as much as possible, with a sex of which they are idolaters’.^13^ This inclination, as so many, would be caused less by education than by an inborn and lifelong ‘physical constitution, by a certain disposition of the organs of the individual’.^14^ Moreover, were an individual so disposed, cross-dressing ‘operates delightfully on his nervous system and on his delicate being’. This fairly unique work challenged not criminalisation (local ordinances targeting crossdressing were in place in France) but popular psychiatrisation, of a ‘mania to assimilate to women […] that some austere censors call real madness’ but that ‘does not change in the slightest [the cross-dresser’s] intellectual faculties’.^15^

Ample evidence shows that nineteenth-century ‘sodomites’ before Karl Heinrich Ulrichs, Ulrichs himself, and the *urnings* he wrote about, often fell subject to this prevailing nosology of monomaniac, ‘fixed ideas’ concerning sex. Ulrichs was mock-diagnosed with ‘gynandromania’, for instance. Itself a conceptual *idée fixe* under heavy fire by the early 1850s, the sponsoring notion of *monomania* had seeped deep into European popular-scientific concepts of mental health, and it is thus that male cross-dressing tended to be classified throughout the latter half of the century: as a man’s *étrange monomanie de se déguiser en femme*, in the late-1880s words of a retired Parisian *chef de la Sûreté.* ^16^ Americans had been comparably calling it *gynomania* since 1877.

Crucially, the nosological nestling of cross-‘sex’ identity among other melancholic ‘fixed ideas of transformation’, including lycanthropy, ubiquitous already by the end of the eighteenth century, remained largely bereft of substantive aetiological attention deep into the nineteenth. This was so despite ubiquitous nosological interest and despite multiple pertinent cases known to Pinel, Esquirol, and their students, including François Leuret. The 1880s, however, saw a wealth of theoretical approaches. The best-known example of this aetiological turn of the fixation of sex *qua* idea – *qua* concept of self – was Richard von Krafft-Ebing’s reception of Carl Westphal’s convoluted, 1869 concept of ‘contrary sexual feeling’ (*conträre Sexualempfindung*). The notion conflated what to many had hitherto seemed distinct phenomena: (homo)sexual orientation and (cross)sexed behaviour. Engagements remained brief during the 1870s but became more ambitious during the next decade. Krafft-Ebing’s concept of *eviration* referred to a putative ‘second stage’ of ‘acquired contrary sexual instinct’ entailing ‘deep and lasting transformations of the psychical personality’. Ultimate – third and fourth – stages of ‘psychosexual degeneration’ in both supposedly acquired and inborn *Konträrsexualismus* entailed delusional – ‘paranoid’ – investments in a ‘sexual’ (*geschlechtliche*) transmutation. This schema connected Westphal’s essentially descriptive approach to gender variance with Bénédict Morel’s degeneration theory. Like multiple contemporaries, Krafft-Ebing sought to reconcile established nosological notions of ‘delusion of sex’ with Westphal’s new formulation of ‘sexual inversion’: a confrontation of early medical notions of *Geschlecht*/*sexe* as *idea* that could be either correct or singularly mistaken (a particular monomania), with new ones of ‘sex’ as a specific and symptomatic awareness or *sense of self* (*Geschlechtsempfindung*). Krafft-Ebing spelt out in 1892 that this new sense of *Sexualempfindung/Geschlechtsempfindung* admitted no overlap with earlier notions of ‘dynamic hermaphroditism’, which hitherto had explained behavioural-psychological variance as the lowest, non-genital and indeed largely behavioural, grade of intersexuality.^17^ Atypical sex identity, in other words, had become a discrete ‘psychosexual’ symptom, with the question of its somato-psychic aetiology an open-ended one.

Contextualised below, Krafft-Ebing’s quadripartite stage model of sexual inversion was the fruit of protracted nosological deliberation. Culminating in 1891, initially as a three-stage model for ‘delusion of sexual transformation’, it entered his *Psychopathia sexualis* only in the subsequent, seventh (1892) edition and, hence, its first English translation. This typology has been considered a baffling moment in pre-Hirschfeldian trans/gender medicine. The here projected continuum between still reversible bi-/homosexual orientation and irreversible sex/gender ‘delusion’ indeed deserves more historical interpretation than has been offered.^18^ Cross-dressing urges here remained what they had been to Westphal, a functional *signum degenerationis* within the purview of *conträre Sexualempfindung.* But questions of how the self was felt or thought to be, specifically how sensations of the sexed/sexual body were being perceived, interpreted, and assimilated to a sense of self, now became much more explicitly problematic, even to some extent oriented around clinical observation. Moreover, differential diagnosis became a more circumspect process. For instance, cross-dressing possibly signified ‘clothing fetishism’, a category specified by Krafft-Ebing in 1889.

Differential-diagnostic problems such as these significantly outlived Krafft-Ebing. At the same time, his stage-concept of sexual inversion proved one of the most durable clinical responses to non-cisgender identification. As late as 1965, we encounter the assertion in a leading American psychiatry journal that *psychosexual inversion* entailed ‘a spectrum of disorders, from mild effeminacy to homosexuality, transvestism, and finally transsexualism, each representing a more extreme form, and often including the previous manifestation’.^19^ This sustained traction, despite Hirschfeld’s intervening ‘sexual intermediacy’ model, is remarkable, knowing that Krafft-Ebing’s empirical basis remained small, and was more than anything the result of a protracted process of bricolage and literature review, during seven years during which *Psychopathia* saw a new expanded edition every year. Furthermore, ‘sexual pathology’ was long only prefacing anything we could call a developmental psychology of gender identity (as might be ascribed, at the earliest, to Alfred Adler). Krafft-Ebing’s explicit qualification of one’s ‘sex-feeling (awareness of a particular gender identity as a man or woman)’ as an ‘important mental characteristic’ only appeared in 1901, and in subsequent editions of *Psychopathia.* ^20^ Only a few authors had hitherto reflected on the development of this sense of sex-consciousness.^21^ Yet Krafft-Ebing was hardly the first nosologist grappling with the *psychological* problem of sex-of-self after Westphal, and his late-1880s interventions at this point need to be seen in light of a variety of contemporaneous sources both cited and uncited by Krafft-Ebing, specifically in light of the nosological status quo as sketched above. Two authors illustrate a wider emergence of what may be called theoretical gender medicine in its own right: Théodule-Armand Ribot and Rudolph Arndt. In the section below, I briefly sketch the historical context for this emergence and discuss both authors in subsequent sections, before asking how Krafft-Ebing fits into this wider context.

## ‘Delusional sex’: from nosology to aetiology

Writing in 1884, Eugène Gley complained about the absence of psychology as a guiding frame for, and, in turn, an unacknowledged key beneficiary of, sexual psychopathological studies, whose ‘sole purpose thus often seems to be to fill nosological frameworks’.^22^ Specifically, if the (*instinctive*) *monomanias* are interconnected, somehow, as degrees or differential outcomes of degeneration and idiosyncratic weaknesses or susceptibilities, each degenerate allowed a unique glimpse of part of the mental mechanism. The sexual instinct being an ostensibly simple faculty, the pervert thus seemed an excellent inroad to psychology. Clinical sex researchers held an important key to nosological reform, well surpassing the new scope of *sexualité.* Gley hinted at fetishism as a developmental obsession, for instance, and like others including Ludwig Kirn, alluded to a stage-model of *hermaphroditisme moral* – quite likely an inspiration for Krafft-Ebing’s later stage-thinking in such terms as *psychosexuale Hermaphrodisie* (‘intersexed’ bisexual orientation). Descriptive sexual psychopathology had come to a dead end, failing at a clinically satisfactory and evidence-based aetiology, and failing to summon or moderate a general psychophysiology of ‘sexuality’ (*Geschlechtlichkeit*; *sexualité*). Franz Joseph Gall’s cerebellar theory of ‘amativeness’ (stipulating an ‘*Organ der Geschlechtsempfindung*’ or ‘organ of gender’ as some phrenologists had it), Paul Moreau’s recent notion of *sens génital*, and Westphal’s unpacked concept of *Geschlechtsempfindung* had all lacked a credible anatomical basis and, more importantly, psychological acumen.

The same can be said about centuries of proto-endocrinological deliberation about the masculinising effects on the brain by reabsorbed semen, a theory that by the 1880s had been sidelined by equally unempirical nervous reflex theories of sexual physiology. Rare attempts at a discriminating neuroanatomy of sexual psychopathology, such as by Valentin Magnan in 1885, did not extend to gender self-identification. During the 1880s, unsurprisingly, Westphal’s concept of ‘sexual inversion’ was increasingly understood to introduce more questions regarding sexual psychology than answers. As a reviewer of Julien Chevalier’s monograph on the subject opined, the second edition of which declared sexual inversion a ‘disorder of identity/personhood’ (*une maladie de la personnalité*), inferences on *inversion psychique* and *sexualité psychique* risked being ‘superimposed on clinical observation and [thus] to bring the facts into line with the doctrine’ rather than vice versa.^23^ The ‘absurdity in the fundamental idea of selfhood’ observed in some ‘inverts’ now posed substantive questions concerning classification, specifically of psychopathy.^24^

From the mid-eighteenth century, considerable attention had been given to aetiology specific to various ‘fixed ideas’ within the sexual sphere: ‘imaginary spermatorrhea’, ‘syphilophobia’, and impotence attributed to magic spells. As discussed, ‘transformation of sex’ was among these various nosological innovations. All of these ‘monomanias’ pertained to radical fears or doubts concerning sex role performance; indeed, all were widely associated with suicidality. Sexual physiology and behavioural neuropsychiatry presented competing explanatory frames for these problems. In 1832, Basel physician-theologian Ernst Joseph Gustav de Valenti (1794–1871) illustratively proposed that even pederasty, perhaps inborn but certainly among the nervous disorders (*Nervenübeln*), betrayed a complex affliction: ‘a monomania of the sexual instinct combined with simultaneous monomania of the psychic sexual love, which transfers sexual lust and sexual love from woman to man […] a true illness, a partial mania.’^25^ Follow-up was minimal. In 1845, Wilhelm Griesinger would extensively mock as unscientific a treatise by De Valenti exploring the relation between madness and sin. But remarkably, de Valenti also anticipated Ulrichs’ notion of mental hermaphroditism. *Tribadismus* was the affliction of the *Mannjungfern*, ‘living withdrawn and solitary, and in remarkably close intimacy with a female person. There are indeed certain hermaphrodite natures [*Zwitternaturen*] which, both through the structure of the genital parts and through the special psychological and moral physiognomy, have made it very difficult for some to develop a certain position in relation to one sex or the other.’^26^

Phrenology presented the most likely arena for arbitration on this point of position-taking, and it was still to phrenology that mid-century alienists deliberating on ‘sex transformation delusion’ turned, however fruitlessly, when asking, ‘To the lesion of what fundamental faculty can we relate such a [‘transformation’] delirium?’^27^ Brierre de Boismont here seems to echo Achille-Louis Foville, who wondered about this precise question already in 1829: ‘To the lesion of what fundamental faculty corresponds the delirium of a man who thinks he has changed into a woman, *and vice versa* […]?’^28^ Franz Joseph Gall (1758–1828) had been interested in ‘partial manias’ from circa 1801 but failed to tackle the problems of sexual and gender diversity head-on. Gall seemingly only once mentioned the scenario ‘when a man believes himself transformed into a woman, a wolf, etc.’, in a passage meant to affirm ‘the correspondence of partial alienations or monomanias with the plurality of cerebral organs, and their partial suffering’.^29^ Research into purported defective femininity/masculinity, however, remained focused on the ‘organ of parental affection’ in women incarcerated for infanticide and those affected with pseudocyesis.

In 1828, Broussais connected monomanias rather to ‘certain changes that have taken place in the [bodily] functions’: ‘This is how a madman rendered impotent by masturbation imagines himself transformed into a woman and wants to take on the [corresponding] tone [of voice] and costume.’^30^ Compromised sexual functions supposedly tricked the mind into the idea of a ‘sex’ switch. In his much translated textbook first published in 1845, Griesinger comparably posited with reference to Claude-François Lallemand that ‘sex change delusion’ was ‘by no means confined specifically to melancholics but may be developed during the course of this disease, and in many cases appears connected with a disease of the genital organs in which the sexual sensations disappear’.^31^ Lallemand’s case, borrowed from André-Pamphyle-Hippolyre Rech (1793–1853), was a ‘man who believed himself to be a girl’ and on autopsy was found to have their testicular functions ‘abolished since the ejaculatory ducts were atrophied and obliterated.’ Lallemand inferred that ‘If this singular alteration of the genital organs were not the cause of the patient’s derangement, it must at least have influenced its peculiar character’. The undisclosed experience of other authors appeared similar, suggesting a somatosensory genesis of partial delirium specific to its sexual content. A student of Esquirol known for his progressive work on humane treatment of the insane, alienist and former asylum physician Maximien Parchappe de Vinay (1800–1866), seemed to fully confirm Lallemand’s inference:

Certain patients, in whom the impulsive solicitations of physical love are lacking, and consequently the actual organic expressions of the need for union of the sexes, find in this state, of which they are aware, the basis of a conviction of impotence; which reacts to the whole life of the soul, which can remain, for a longer or shorter time, as the exclusive symptom of psychic disturbance, and which can also enter, either primitively or concurrently, as a partial element, into a more or less extensive delirium. It is to this real diminution of the natural impulses that can often be referred, as a point of departure, the fixed ideas and the delirious conceptions of madmen who believe themselves impotent, eunuchs, or who imagine themselves to have changed sex. […] I had the opportunity to observe, in a patient whose melancholy proceeded to dementia, the coincidence of the fixed idea of sex change [idée fixe du changement de sexe], with a state of atrophy of the genital parts, the inertia of which was moreover invoked by the patient as the sure sign of his metamorphosis.^32^

This line of reasoning provides an important backdrop to 1880s interests in *sexualité*, which evidence an increasingly wider variety of speculations, still mostly theoretical. In mid-1870s lectures, prominent French psychiatrist Benjamin Ball (1833–1893) provisionally tied the Hippocratic ‘Scythian malady’ to the supposed mental corollaries of genital hyperesthesia, for instance.^33^ German psychiatrist Heinrich Neumann had tied it rather to male genital anaesthesia.^34^ Parisian alienist Evariste Marandon de Montyel (1851–1908) proceeded beyond late eighteenth-century associations with masturbation, by affirming a role for spermatorrhea. Semen loss would produce a *variété vésanique*, however one whose specific symptoms were culturally tinged: a *folie séminale à couleur locale* – ‘seminal mania’ in William Alexander Hammond’s 1882 translation.^35^ Hammond himself reaffirmed a hitherto well-established testicular atrophy theory, emboldened by his analogy of such an ailment he reported having observed, in the early 1850s, among Puebloan *mujerados.* Furthermore, various delusional states and irrational fears with some gender or genital theme were recorded by the earliest commentators on *conträre Sexualempfindung* during the last decades of the century: syphilophobia, gynophobia, male pseudocyesis. An 1882 Italian report often cited in the 1880s literature on ‘sexual perversion’ was that of an apparent cross-sex delusion in a gynecomastic man, a report that took aboard the possible relevance of the famed Scythian disease as well as the recent concept of sexual inversion.^36^

The foregoing significantly qualified blanket verdicts of *intellectual* (rather than *instinctive*) *monomania*: atypical adoption of sex roles was now a culture-relative and clinically heterogenous affair. Medical perspectives on male gender-crossing had in fact long been relativized by ethnological perspectives on this ‘Scythian-like’ phenomenon. A word had even been coined for it: ‘Enareanism (if I may so term it)’.^37^ Though he himself never discussed the ‘feminine malady’, in a footnote Westphal acknowledged an association of his own December 1868 oral paper with various phenomena widely correlated in earlier literature: the Scythian *anarieîs* (the ‘unmanned’), native American cross-dressing, and the Chevalier d’Éon. This unpacked reference is to pioneering physician-ethnologist Adolf Bastian, the founding editor of the *Zeitschrift für Ethnologie*^,^ writing in its maiden volume.^38^ Bastian delivered some of this material apropos Westphal’s oral presentation at the December 15, 1868 meeting of the *Berliner medicinisch-psychologische Gesellschaft*, which he attended. Earlier in 1868 Bastian had advanced a tentative, if woefully unprocessed, ‘comparative psychological’ frame for gender-crossings, under the heading of ‘the pathology of possession’.^39^ Bastian had still earlier categorized species- and ‘sex’-switching as *insania metamorphosis*, quoting a psychiatry textbook.^40^ However, these occasional ethnopsychiatric allusions had remained woefully unelaborated.

Where Pinel’s *manie*/Esquirol’s *monomanie* frame for ‘sex change delusions’ had pinpointed a singular blind spot in rationality, Westphal stressed an observed awareness on the part of the patient of ‘the morbidity of the phenomenon’ of inborn sexual inversion. This nosologically required awareness was problematic given that, in the perception of many, it was only in Westphal’s text that such a verdict began to be insisted upon by alienists themselves. Most indicative of a move here from psychiatric conditions defined by discrete symptoms toward a more compounded, aetiological concept of sex-gender intermediacy, Westphal problematized the verdict of ‘periodic mania’ with regard to his single cross-dresser case, which had appeared in a report written by a penitentiary inspector.^41^ The patient’s own terminology evidently only included *Drang* (urge) and *Trieb* (drive), Westphal observed, though without problematizing these terms. Westphal himself spoke interchangeably about *Drang, Tendenz, Verlangen, Trieb, Hang.* He was at this point clearly resisting Pinellian/Esquirollian readings of non-cis gender identification: here was not a ‘partial alienation’ but a strong desire urging the patient to relate to themselves and to the world in ways they realized were contrary to gross-anatomical expectation. A reviewer for the *Annales médico-psychologiques* (Auguste Chatelain, 1838–1923) did not pause at what he glossed ‘*manie de porter des habits de femme…singulière manie*’ – but clearly these dated designations now required more caution.^42^

Antique and new ideas were seen side by side during the closing decades of the century. By 1888, the Scythian malady was still widely cited as only one of a variety of symptomatic instalments of ‘identity confusion’ (*Personenverwechslung*), even associated with a FtM case presented by Konrad Alt (notably in a journal co-edited by Krafft-Ebing).^43^ Classical scholar Wilhelm Heinrich Roscher (1845–1923) thought likewise, fully agreeing with an opinion then already over a century old, and also considered by Arndt: here was a ‘therianthropy-like pathological phenomenon’.^44^ However, even authors uninformed of the new diagnostic slot of *sexual inversion* leaned toward reframing: aetiology and development now became central objectives. French physician Ernest Martin (1830–1897) wrote in 1880 that here was ‘a monstrosity that would undoubtedly be difficult to find in our time but which existed in the time of Hippocrates because he gives a very precise description of it’.^45^ But in tune with many contemporaneous voices, even Martin actively pursued modern analogies (he continued with a long account of d’Éon) and even threw in a developmental-nosological speculation regarding such ‘monstrous’, but not ‘hermaphroditic’, cases: ‘During development their constitution did not follow a clearly determined sexual direction; it remained hesitant and it offers the spectacle of a singular whole for which it is difficult to give a physiological explanation unless it is considered as an atavistic manifestation which would be based on the primitive androgyny [*androgynisme*] of Man.’^46^ Psychological ‘sex’-switching, then, seemed in a particular class of its own meriting developmental perspectives.

## ‘Metamorphosis from above not from below’: Ribot

While sexual preoccupations and fears invited psychiatric verdicts already throughout the eighteenth century, concepts of sexual disorder took a progressively psychological turn during the late 1870s and 1880s, especially in France.^47^ The psychology of selfhood saw widespread attention, often with reference to clinical problems related to identity, self-perception, and self-awareness. One’s notion of one’s sex (*sexe comme idée*) now spoke to increasingly general questions of the fixity of self-identity (*notion/sens intime de la personnalité, notion/conscience du moi*). ‘Errors’ or ‘alienations’ of self-identity (*erreurs/alienations de la personnalité*) prominently included multiple identities (*personnalités multiples et coexistantes*) seen in hysterics, and fictive identities (*personnalités fictives*) brought about by suggestion.^48^ German philosophers and experimental psychologists before Westphal had been using terms such as *Selbstempfindung/Selbstgefühl* (also called *Gemeingefühl, Allgemeingefühl*) which in psychiatry came to mean a self-consciousness (*Selbstbewusstsein*) or self-concept (*Vorstellungscomplexe des Ich*). Such faculties were seen to be at the mercy of, and thus at risk of being deceived by, the senses: by pleasure and displeasure (*Lust*/*Unlust*) and their potential excesses. The Self, the *Me*, was dissected more elaborately and explicitly than before, and although bereft of a clear-cut concept of ‘gender identity’, multiple authors struggled with the now psychological-developmental questions of how sexual instinct, sexed bodies, and sexual functioning were to fit into this psychological Me. We have William James, for instance, insisting that clothes were quintessential ‘*constituents* of the me’, the outer part of the ‘material Self,’ a point of acute relevance to Hirschfeld’s *Transvestiten.* ^49^

James stopped short of explicitly reifying sexed appearance and sex role, however. More remarkable at this point is mid-1880s work by Théodule-Armand Ribot (1839–1916), founder of experimental psychology in France and one of a number of contemporaneous French authors to reflect on identity-selfhood, or *personnalité.* ^50^ Ribot has been considered one of the first French experimental psychologists with an interest in sexuality, which, as Sylvie Chaperon has argued, proceeded mostly through psychopathology. He never offered cases, but with François Leuret’s of the 1830s in mind, in 1884, Ribot provided a primarily moral-cerebral approach to specifically sex-of-self crossings.^51^ This was evolving insight, judging from a very brief mention of cross-*sexe* identification already in 1880, building up to a hitherto rare developmental frame for Westphal’s *sexualité contraire*: development of the sex character (*caractère sexuel*) of the mental apparatus.^52^ ‘This development of an organ and of its functions, with their train of instincts, imaginings, feelings, sentiments, and ideas, has produced in the neuter personality of the child a differentiation – has made of it a male or female Me [*un moi mâle ou femelle*], in the complete sense of the term’. This bare postulation of developmental psychosexuality was to guide extrapolations from physiology to ‘exceptional and morbid cases’, including the mental condition of the intersex individual and the eunuch being ‘emasculated morally as well as physically’ – or ‘mutilated in mind as in body’, in the words of Britain’s leading mental pathologist Henry Maudsley.^53^ But even Maudsley, in a single unpacked sentence, had also mentioned something else, as if to support his wider claim of ‘the intimate and essential sympathy between the brain as a mental organ and other organs of the body’: ‘And there is certainly a characteristic variety of insanity caused by self-abuse, which makes the patient very much like a eunuch in character’.^54^ This may or may not refer to a prevailing French interpretation of Hp. *Aer.* 22 seeing feminisation as a corollary of onanism/spermatorrhea, though this outcome clearly differed from the more characteristic, florid, and lethal ‘mental derangement’ by means of self-abuse.^55^ Regardless, these phenomena in clinical psychology Ribot considered suggestive of the outstanding heuristic value of sexual disturbances in conceptualizing the larger, hitherto elusive phenomenon of selfhood: ‘For if *personnalité* [i.e. sense of personhood, identity, sense of self] is a compound, varying according to its constituent elements, a change in the sexual instincts will change it, a perversion will pervert it, an inversion will invert it [*une interversion l’intervertira*]; and this is exactly what takes place.’

Clinical experience suggested an additional, reverse causality, however. For in ‘sex change delusion’ cases such as Leuret’s, ‘the starting point of the [gender/psychosexual] metamorphosis is to be sought elsewhere: it must be found in the cerebrospinal organ.’ As would not much later Krafft-Ebing among others, Ribot ended up postulating neurological ‘centres from which psychic incitements are sent out to the sexual organs to put them in action […] the cerebral, and consequently the psychic, representatives of the sexual organs’.^56^ This cerebral seat of sexuality, then, would explain cases of ‘metamorphosis from above not from below’: that seen in known cases of a ‘cerebral disorder of unknown character (a woman supposing herself to be a man, or *vice versa*) whence results a fixed erroneous state of consciousness.’ This consciousness then *seeks its own completion*, radiating widely to ‘the feelings, the ways, the speech, the dress of the imaginary sex.’ Cross-*sexe* identification and desire here became one of the ‘cognitive deviations of identity’ (*déviations intellectuelles de la personnalité*). The ‘psychological monstrosity’ of *sexual inversion*, by comparison, would be an entirely different and indeed larger problem: precisely that of the *lack* of parity in character between body and soul, where, in other words, consciousness does *not* ‘tend to complete itself’ and rather produces homosexuality. Psychology, here, would for the moment have to admit its ignorance, Ribot insisted. Degenerationists, for instance, could indeed not explain ‘why degeneration takes this [sexual inversion] form and not another’; maybe ‘the mystery is to be sought in the multiple elements of heredity, in the complex play of the conflicting male and female elements’.^57^

Ribot never set out to unravel the mystery laid out here. In evaluating his 1880s contributions, it is notable that he never provided a critical review of the available literature on the psychological sequelae of castration, for instance. Castration had been a minor theme in contemporaneous psychiatry that spoke most directly to this theorising. One example is a Russian discussion of the psychological sequelae and psychiatric profile of self-castration as practised by the Russian Skoptsy, translated into German in 1876.^58^ Not unlike 1860s reactions to Ulrichs’ concept of ‘mental hermaphroditism’, the alleged ‘proselytizing’ and ‘propaganda’ of the afflicted (the sectarian self-afflicted, more precisely) proved all-too-easy fodder for psychiatrisation. Illustratively, the ostensible difference between eunuchs and the cross-dressing pseudo-eunuchs of Hippocratic fame proved assets in criticisms of Krafft-Ebing’s *Anlagetheorie* of sexual inversion and its appeal to notions of bisexual constitution. For while an intimate correspondence would have to be assumed between gonads and brain, castration in the usual sense did not activate any latent potentiality for a ‘new sexuality’, let alone a full *sexe*-identity switch, August Cramer noted in 1897: ‘The eunuch is indeed a silly and effeminate fellow and makes a womanly impression, but he does not feel like a woman; he develops no sexual inclinations at all, least of all homosexual ones.’^59^ At the same time, homosexuality would not be *inversion* but perhaps a mere ‘product of the imagination,’ and as such fully compatible with both physical and mental health, even beyond the developmental years. Where this intervention into nosology left cross-gender identification, Cramer did not develop, however.

## *Selbstempfindung* and *Geschlechtsempfindung*: Arndt

It was under the early modern nosological heading of ‘delusion of metamorphosis’ that an early sense of gender identity acquired definitive contours in Krafft-Ebing’s nosological thinking. Three short borrowed ‘sex-change delusion’ cases came to speak to his classification. These cases frustrated Krafft-Ebing’s aetiological interests, however. Ostensibly for this reason, they were still discussed in full in *Psychopathia*’s ninth (1894) edition but only cited from the tenth (1898) onward. Henceforward, only Krafft-Ebing’s own cases were integrally discussed (something that perusers of the Rebman translations, based on the tenth and twelfth editions, risk overlooking). They illustrate the ongoing clinical reasoning in which Krafft-Ebing was intervening. Krafft-Ebing quoted a case by Rudolph Arndt (1835–1900) in full, as one of the very few, besides ‘two cases known to Esquirol’, to exemplify *metamorphosis sexualis paranoica*, complaining that little could be learned from any prior case about possible developmental factors – Krafft-Ebing’s central concern.^60^ Krafft-Ebing had read Arndt’s work when it came out and had also read the relevant passage: in a book review for the *Zeitschrift für die gesamte Strafrechtswissenschaft*, Krafft-Ebing specifically pointed that passage out as having forensic relevance. In this sole contemporary description of cross-sex identification (by a transmasculine person) beyond his own that Krafft-Ebing was able to cite as late as 1891, the condition had still been assimilated with the madness of lycanthropy and other forms of *mania metamorphosis.* ^61^ But Arndt’s take was more creative than Krafft-Ebing suggested.

A bold and comprehensive intervention into nosology, Arndt’s book proposed a new system of classification for mental disorders such as would reflect a more empirical, scientific basis in nervous excitation and muscle contraction – ‘an attempt, too little noted’ as another reviewer of his work would write in the 1888 maiden volume of the *American Journal of Psychology.* Where it *was* picked up, it scored mixed reviews. Arndt had been appointed in 1873 as *Extraordinarius* and in 1875 as independent director of the Greifswald Communal Irren-Heil-Anstalt, a step formalising the independence from internal medicine of the speciality of psychiatry at the site. Here, Arndt had had the opportunity to observe ‘a middle-aged woman who thought she was a man and carried herself [*sic*] accordingly.’ Patient ‘always felt something like this; however, the clarity of this only came to me later’. Moreover, ‘The man who is supposed to be my [patient’s] husband is not a real man at all. I conceived my children myself.’^62^ At this point, Arndt interpreted the still new-ish phrase of *conträre Sexualempfindung* as a being drawn ‘to the behaviour of the opposite sex’ purely out of a *paresthesia* ‘which in the ego [develops] into delusions or insanity’ (psychosis, we would say, or *paranoia*, in Krafft-Ebing’s later terminology), demanding conformity to the new self-image. In a remarkable passage, moreover, Arndt specifically asserted the privileged developmental importance to *Selbstempfindung* (with attendant *Selbstgefühle*) of the *Geschlechtsempfindung*, or sense of sex: ‘the main weight for the development and transformation of self-awareness and self-feeling may be ascribed primarily to the sexual sensations and the processes underlying them’. What makes a man or a woman? The answer would have to be the differentiation that comes with sexual development:

[t]he complete change of the whole personality, the powerful development of the body, above all the skeleton and the muscles in the man, the voluptuous forms in the woman, the daily increased experience of becoming more and more efficient, and above all the ever more and more strongly developing feeling of being a man, being a woman – these lead to it. […] Ultimately, therefore, the man, the woman, is what their sex glands are. […] The associated paresthesias, parathymias, show themselves above all in the wrong feeling of oneself, in Westphal’s so-called contrary sexual sensation, in higher degrees of which the man feels as a woman, the woman as a man, and this, according to his wrong self-feeling [*verkehrten Selbstgefühle*] also touched by everything […].^63^

Arndt thus reasserted the central clinical pertinence of *contrary sexual feeling* to not only the wider problem of the *paresthesias* and attendant ‘identity disorders’ but also to the developmental aspects of normal ‘self-feeling’. Cited allusion to progression (‘higher degrees’), which inverted such a sense present in Westphal’s text, anticipated or perhaps directly inspired Krafft-Ebing’s initial (1891) stage-model.

*Paraesthesia sexualis* (*Parästhesie der Geschlechtsempfindung*) is jargon notably adopted by Krafft-Ebing three years later to cover all sexual perversion and inversion.^64^ Terminology at this point clearly reflects different nosological routes, announced by two new uses for the term *paresthesia*: Arndt’s physiological frame for ‘identity disorders’ (with a purportedly prominent sexual dimension and aetiology) and Krafft-Ebing’s *psychopathia sexualis* (featuring ‘paranoid’ conceptions of social-corporeal transformation, as sexual inversion in optima forma).

## Krafft-Ebing: *Geschlecht* and paranoia

Arndt’s short case description remarkably left out the aetiological leads he himself asserted and which Krafft-Ebing considered pivotal. Krafft-Ebing can thus properly be called the first to have given what would become a third-stage ‘acquired’ transitional variety its single, elaborate autobiographical account – ‘*ein Unicum*’.^65^ Similar to Ribot, Krafft-Ebing’s eventual call of *metamorphosis sexualis paranoica* rearticulated a long-established notion of lasting delusion of ‘sex’ transformation, granting it a clinical kinship with other sex-related species of ‘paranoia’ Krafft-Ebing had distinguished since his earliest days in medicine. This entailed a new nosological embedding and a new aetiological profiling, beyond Westphal, of eighteenth-century notions of the *Wahn der Geschlechtsverwandlung* – or as the 1895 French translation of *Psychopathia* (from the English, by Émile Laurent and Sigismond Csapo) had it, *la monomanie de la métamorphose sexuelle.* Krafft-Ebing’s most extensive 1890 autobiographical case of *metamorphosis*, by a physician, has been marked as one of the first ones on record, and one much-reinterpreted, as exhaustively studied by Julien Fischer.^66^ What interests me here is that the case was understood early on as indicative of the particular aetiological problem presented by the emergent nuance of ‘sex’ in strict terms of ideation, and sex in the narrator’s own consistent terms of *Gefühl* or *Zwangsgefühl* guiding their ideas of self. A new clash over narrative authenticity and delusional content had been emerging at this point, complicated by the narrator being a colleague physician. One reviewer illustratively preferred to trace the content of the ‘delirium’ of cross-’sex’ awareness back to the author’s intimate medical knowledge: ‘No one other than he [*sic*] who by profession was acquainted with all the physiology and pathology of woman would have been capable of a delirium similar to that which he [*sic*] presents.’^67^

As seen, appraisal of the ‘*Wahn das Geschlecht geändert zu haben*’ as an instance of *melancholia*/*mania metamorphosis* long predates Krafft-Ebing’s earliest work, going back to the middle of the eighteenth century and ending up as a nosological commonplace in Krafft-Ebing’s youth, during the middle of the nineteenth.^68^ Relevance of nosology to legal medicine within the new frame of sexual inversion – ‘psychosexual degeneration’ – became increasingly apparent. A reviewer of the eighth edition of *Psychopathia sexualis* praised Krafft-Ebing on this particular belated elaboration of Westphal: the seeming deterioration, or *decursus*, of the invert from the habituated or occasional pederast (*gezüchtete Päderast*) to the *Wahn vollkommener Geschlechtsveränderung.* ‘We see this gradual transition in a series of cases in which the same actions [‘coitus-like’ acts] must still be described in the first case as crimes that cannot be excused, and in the latter as expressions of illness by incurable madmen.’^69^

Krafft-Ebing replicated Arndt’s inversion of Westphal’s unelaborated musing that contrary sexual feeling was ‘also simply about the feeling of being alienated from the whole inner being with regard to one’s own sex – as it were a less developed stage of the pathological phenomenon’.^70^ Westphal’s unpacked allusions to ‘progression’, or rather deterioration at this point of symptomatic divergence, had hitherto remained unelaborated, as merits brief review. Simply inverting the allusion, in 1878, Heinrich Schüle, a close colleague and *intimus* of Krafft-Ebing, had captured ‘psychic hermaphroditism’ in terms of ‘delusion of altered personality’ (*Wahn der geänderten Persönlichkeit*) with respect to birth-assigned *Geschlecht*, footnoting the Scythian *anarieîs* to the cryptic characterisation of ‘the delusion of altered personality as the highest allegory of abnormal sexual feeling’.^71^ Schüle recognized both cross-dressing and ‘sex’-crossing as possible manifestations of sexual inversion but shied away from inferring a stage model: ‘Contrary [sexual] feeling can even dominate the experience of self [*Persönlichkeitsgefühl*] of the patient, in such a way that the male patient feels transformed into a woman and the female patient into a man, and each *optima fide* carries out their metamorphosis in clothing and occupation.’^72^ *In the best faith*: patients in question were ‘constitutional defectives’ (*Defectmenschen in ihrer Anlage*), he opined, anticipating Krafft-Ebing’s later parlance of ‘degenerate reaction’ and ‘functional symptoms of degeneration’ (*funktionelle Entartungssymptome*).^73^ Schüle notably also mentioned cross-dressing in his degeneration-based stage-model of hysteria – an emergent differential-diagnostic concern.^74^ Drawing from aforementioned Bastian, one of the founders of developmental psychopathology Hermann Emminghaus (1845–1904) insisted on a cleaner differential diagnosis: ‘From Westphal’s contrary sexual sensation as an ‘innate perversion of sexual feeling with awareness of the pathological nature of the appearance’ one must clearly distinguish the delusion of sex change [*Wahn der Geschlechtsumwandlung*], which occurs not infrequently in the mentally ill’ – telling ‘analogies’ of the latter, again, to include the *anarieîs* and various modern-day indigenous *comparanda.* ^75^ Emminghaus had earlier cited these analogies as the many ‘equivalents’ or ‘parallels’ to be found between primitive psychology, developmental psychology, and mental illness, suggestive of a common structure of stepwise cognitive evolution (*Stufen des Denkens*).^76^ In comparison, Prague psychiatrist Pick characterised the Scythian illness as ‘a side piece to contrary sexual feeling’ but failed to elaborate.^77^ Russian venereologist Tarnowski’s remarks at this point, finally, also remained poorly developed. Under ‘inborn sexual inversion’ Tarnowski spoke only loosely of *Bestreben* or *Neigung dem weiblichen Geschlecht zu ähneln*, writing:

Despite the tendency so clearly expressed to resemble the female sex, the perversity of the sex sense in the cited case [of Elise/Eliza Edwards’ ‘concealed sex’, as discussed by Alfred Swaine Taylor] differs in no way from the type described above of the born passive pederast or so-called ‘Cynedes’. But in addition to such conditions that can be described as intermediate forms [*Mittelformen*], there are other pathological deviations, sometimes weaker, sometimes more pronounced, which form a gradual transition to clear forms of hereditary mental disorders.^78^

In summary, then, during the 1870s and early 1880s, Westphal’s ‘sexual inversion’ was breaking apart along the typological, differential-diagnostic, and comparative lines he himself only faintly hinted at, however resulting in little unanimity.

A brief reconstruction of Krafft-Ebing’s taxonomy demonstrates a gradual buildup of courage in distinguishing clinical variation in terms of progressive underlying pathology. Krafft-Ebing actively worked on psychosexual nosology from 1875 and, like others during the early 1880s, alluded to the idea of a ‘stage’-model for sexual inversion already in 1882.^79^ Case 24 of *Psychopathia*’s 1886 edition only documented passing cross-‘sex’ identification in a ‘congenitally’ sexually inverted woman. In the 1887 and 1888 editions, Krafft-Ebing developed a concept of *effeminatio* (rechristened *eviratio* from 1891) covering, rather, ‘more profound and lasting changes in the psychic personality [i.e. identity, sense of self]’, and typically caused by masturbation. He discussed the concept, seemingly first, at the March 14, 1887, monthly meeting of the *Verein der Aerzte* in Steiermark (Austria).^80^ A patient assigned male at birth experienced ‘a profound change in his character, especially his feelings and tendencies in the sense of a womanly self-identity [*einer weiblich fühlenden Persönlichkeit*]’.^81^ In 1888, Krafft-Ebing elaborated that ‘permanent change of identity’ (*dauernde Umänderung des Persönlichkeitsbewusstseins*) would only be seen in the case of a full-blown ‘paranoid’ condition, and as such would constitute a ‘capstone of all physio-psychic transformations’ (*Schlussstein all der physiopsychischen Umwandlungen*).^82^ This closely followed the progressive nature of *intellectual/systematised insanity* generally entailing, in Emil Kraepelin’s terms, ‘a permanent, profound transformation of the psychic personality, which manifests itself mainly in a pathological perception and processing of external and internal impressions.’^83^ To Krafft-Ebing, the phrases *Metamorphosis sexualis* (as used from 1887) and *Transformatio sexus* (as used from 1888) named a passage from a pre-existent cis-‘sexual’, to cross-‘sex’, identification: one symptomatic dimension of several. An early, implicit outline of his later *Gradstufen*-theory came in 1888. Two *Stufen* became explicitly distinguished in 1890: homosexuality per se and *effemination*, the latter covering all sexed crossings. Only in 1891, these became more formally divided into three consecutive steps in ‘psychosexual degeneration’ (*Etappe[n] auf dem Wege der psychosexualen Entartung … Entwickelungstufe[n]*). These stages were now more explicitly than before situated beyond what is here newly nominated the last outpost of (male) normality: the *gezüchtete Päderast*, perhaps indifferent in his inclinations but who, where homosexually active, still ‘feels himself in an active role corresponding to his real sex’. Normality was now sex role conformity, the degree of pathology now being the extent of role switching. In 1892, this, finally, made the definitive four steps of a now singular ‘somato-psychic transformation process’ (*eines körperlich-seelischen Umwandlungsprocesses*) where a person gradually retires from their natural embodied sex role, beyond mere deviating from the *Sexualrolle* (sexual role-taking: topness/bottomness) natural to their sex.

Krafft-Ebing’s rubric of ‘paranoia’ reflected considerable clinical problematization of people’s *idea of sexed selfhood* since Westphal’s 1868 talk. Krafft-Ebing’s *paranoia* (Esquirol’s *intellectual monomania*) was mid-1880s terminology replacing what in 1883 he, with Kraepelin, still preferred calling ‘*primäre Verrücktheit*’. The general term generally referred to a chronic mental disorder consisting mostly of delusions: fictions based on false premises which progressively organise themselves and end up accepted as incontrovertible facts.^84^ The patient acts as if their delusions were true, and comes to inhabit ‘a world of error and deception’ built around a core misconception. It required of the clinician knowledge of the truth that was sex, and a reconstruction of how, over time, the patient could have drifted from that truth as far as to assert that its arguable opposite was true. The condition would have to be engrafted on a degenerative basis: it betrayed some sort of hereditary taint, often a constitutional neurosis. Full recovery was not an attainable goal, but anamnesis was key to understanding it. Most relevantly here, it provided a lead to general sexual psychology. To Krafft-Ebing, the content of the paranoia betrayed a primordial abnormal development of the entire personality: it expressed a ‘hypertrophy’ of a deep psychological core. Disease developed out of ‘the innermost nucleus of the personality’ or ego (*dem innersten Kern der Persönlichkeit … der charakterologischen Individualität des Kranken*): the unconscious ‘depth of the soul’ (*dem tiefsten Grund ihrer Seele*) where dreamy, romantic aspirations are elaborated into guiding ideas.^85^ The sexual body was not trivial in the causal cascade: castration, puberty, menopause, menstrual and uterine disease, and onanism presented cardinal predisposing moments for melancholy/paranoia, various of Krafft-Ebing’s early texts asserted.^86^ These moments of sexual transformation, however, Krafft-Ebing saw as pristinely and universally *psychological* transformations, requiring a medical-psychological sensibility to psychological diathesis, beyond a focus on changeable ‘sexual sensations’ highlighted by Kraepelin among many others.^87^ The climacteric, which is to say, women’s ‘sexual role (*geschlechtliche Rolle*) being played out […] cuts deeply into the sense of personhood [*Persönlichkeit*], [it] does not go unnoticed in any woman’s consciousness. The loss of those [sexual] feelings, which form a large part of the moral self-concept [*ethische Persönlichkeit*], and are ultimately rooted in sexual sensations, is felt by every woman at least as a defect.’^88^ Various mundane shifts in the sexual biography, then, shared a common, dual ground in changing somatic sensibilities and challenges to the continuity of a sense of socio-sexual selfhood.

Krafft-Ebing’s reasoning here needs to be seen in light of the unacknowledged precedents in clinical-psychological reasoning regarding *mania metamorphosis*, as discussed above. These rendered the condition of ‘progressive melancholy’ of specific psychological interest, as Krafft-Ebing agreed with contemporaries who were citing Griesinger at this point of *délire d’interversion sexuelle.* ^89^ Krafft-Ebing’s ‘paranoia’ variants invariably betrayed a basis in sexual disorder, which the clinical course would clearly demonstrate. *Paranoia (sexualis) masturbatoria* – originally filed under hypochondric-persecutory madness, or *masturbatorische Verrücktheit* – was to account for the slow descent of the young onanist into various ideas of supposedly consequent nervous disease and imaginary social censorship. A continuity here with older German literature on ‘abuse of the sexual function’ (*Geschlechtsmissbrauch*) is evident. Compare, for instance, a lively description of demonomania in an onanist by Rudolf Leubuscher in 1848, and various forms of melancholy attributed to genital disorders (*melancholia uit de organa generationis*), including onanism, by pioneering Dutch psychiatrist Schroeder van der Kolk.^90^ The problem proved a durable one; an increasingly psychological sense of *Masturbantenwahn* is seen in various early twentieth-century texts, for instance. Krafft-Ebing’s take seems crucial for the present discussion, given that from 1886 he named neurasthenia *ex masturbatione* a key proximate cause for acquired ‘homosexual sensation’. From 1889, he added venereophobia (‘hypochondriacal fear of infection from sexual intercourse’) as a possible factor. One of the earliest Dutch cases of an *urning*, published in 1893, was a lifelong onanist whose clinical presentation was largely taken up by that onanist’s acute regret and a guilt-ridden impression that all people in the street knew of his homosexual condition and surely were referring to his person when using corresponding epithets.^91^ Other discrete exemplars of paranoia included *paranoia hysterica*, *sexualis* (including delusional contestations of sexual reputation and delusional jealousy), and *erotica* (erotomania). In their joint bridging of genital disturbance, nervous deterioration, and psychosexual angst, these diagnoses all pinpointed patients’ self-caricaturing within the gendered and generational realms of sexual conformity and reputability. Though never formally extended to a *paranoia Scythica/sexus*, it is indeed under *Verrücktheit/paranoia* that Krafft-Ebing situated some of the melancholic identity ‘transformations’ of old, such as lycanthropy: an established and receptive canvas for his evolving conception of identifications concerning *Geschlecht.*

A chapter on the ‘*Wahn der Geschlechtsverwandlung*’ in the second edition of Krafft-Ebing’s *Neue Forschungen auf dem Gebiet der Psychopathia sexualis* shows a watershed moment at this point of aetiological deliberation on what he here called ‘the awareness of representing a particular gender-personality [*das Bewusstsein, eine bestimmte geschlechtliche Persönlichkeit zu repräsentiren*]’.^92^ This concept formed the basis for purported pathology in its purported end-stage: ‘delusion of really being another person sex-wise’ (‘*Wahn eine andere sexuelle Persönlichkeit wirklich zu sein*’).^93^ In the German literature a virtually unprecedented discursive manoeuvre (except for Arndt, as seen), Krafft-Ebing here briefly conjectured about *normal* gender identity development, which, however brief and ineffective, made it into *Psychopathia*’s subsequent editions.^94^

The core nosological quandary was that this descent into cross-sex identity at once reflected, and betrayed, an underlying ‘degenerative’ ‘feminization’. A degenerate, neuropsychologically ‘feminized’, man is not necessarily wrong in saying that he was, or had been, passing from male, *man*, to female, *woman.* In an 1889 new case interestingly dating back to Krafft-Ebing’s time at Illenau Heilanstalt in the mid-1860s and evidently seen by Heinrich Schüle up until 1872, a complete example is described as a purely psychological resolution: ‘From the time of the *Transformatio sexus*, a new era begins for the patient. In memory, he conceives his previous identity [*Persönlichkeit*] as that of a cousin’.^95^ Yet as many nineteenth-century cases, in this case description and an additional contemporaneous one introduced in 1890, various psychotic elements were noted: female identity, but suggested by voices; preceded by masturbation, but more interestingly by *paranoia masturbatoria persecutoria*; all next to clinically observed delusions and hallucinations seemingly unrelated to sex/gender.^96^

## Coda: between paranoia and transvestitism

An 1893 paper that seemed to confirm Krafft-Ebing’s 1891 nosological suggestion did not cite Krafft-Ebing, illustrating that his work took time to disseminate and to dominate 1890s clinical thinking, and that it articulated theoretical elements independently pondered by many others. In a paper presented June 7, 1893, to the American Medico-Psychological Association, medical superintendent at the Eastern Michigan Asylum and lecturer in mental diseases Colonel B. Burr (1856–1931) discussed a female long-term inpatient with a rich spectrum of delusions (royal heritage, pseudocyesis, poisoning), a two-year unstable insistence on having a male identity beginning years into admission, as well as an apparent sex operation delusion (‘asserting that many years ago an operation was made with the effect of unsexing her’).^97^ The ‘paranoia’ in this case was considered to have arisen with the growth of a uterine fibroid tumour (explaining pseudocyesis, though not the coexisting cross-gender identification), and to have attenuated somewhat after its removal, which effectively meant a hysterectomy. Burr evidently was most impressed by the durable ‘delusions of change in sex,’ and briefly sought to make sense of it by reaching out to Ribot, not Krafft-Ebing – despite Burr’s choice of diagnostic terms: ‘If, as Ribot contends, ‘the personality results from two fundamental factors – the bodily constitution, with its tendencies and feelings, and the memory,’ we must, I think, seek for the genesis of this delusion in the changed sensations arising from the sexual organs in consequence of its [neoplastic] presence’.^98^ ‘Sex’ self-identity assertions could betray a ‘systematized delusion’ as part of a heterogeneous spectrum of fixed ideas, and not necessarily as an advanced stage in sexual inversion or as paired with same-sex erotic interests.

A proper differential-diagnostic moment had emerged, in other words. Krafft-Ebing’s very limited experience admitted two seemingly separate types of ‘degenerative’ disorder related to ‘sense-of-sex’: sexual inversion and *original paranoia.* Yet his integration of either within the prevailing nosological frame of *Wahnsinn*/paranoia remained curiously minimal. *Psychopathia*’s brief section on sexual forms of *paranoia* never cross-referenced ‘sexual inversion’, while even the last (the posthumous seventh, 1903) edition of his general psychiatry textbook did not mention ‘*Metamorphosis sexualis*’. ‘Paranoia’ pertaining to *Geschlecht*, then, was of a symptomatic, noncanonical kind, and, one could easily object, another class of disorders altogether. Munich specialist in nervous diseases Leopold Löwenfeld (1847–1923) illustratively dismissed such cases from the realm of sexual disorders: ‘Since this is a form of paranoia, we can no longer consider the cases in question to belong here, as we are merely dealing with the anomalies of the sex drive.’^99^

Whether notions of *Geschlecht* were tethered to or followed from *Sexualempfindung* was now a proper differential-diagnostic question, puzzling at least some physicians already at the moment Krafft-Ebing was only finalising his thinking on the matter. In 1891, neurologist-psychiatrist Christian Roller (c.1843–1897), seemingly uninformed by Krafft-Ebing’s *Neue Forschungen*, presented three ‘cases of contrary sexual ideas’ (*Fälle von conträrer Sexualvorstellung*), stressing that these cases ‘do not show the actual contrary sexual sensation [by which he means *homosexuality*; but rather] the delusion of belonging to the opposite sex’:^100^

The delusion of belonging to the opposite sex […] deserves special consideration because it represents a reversal of a fundamental group of ideas. The delusion in question is, to put it this way, not a psychological but a physiological error of a pathological nature, and one of an elementary nature at that. This delusion is obviously of a different nature than many other delusions and perhaps also of a different pathogenetic nature. While the process occurs, resp. expresses itself, primarily in the area of imagination, the corresponding sensations are also present in these patients in a more or less developed way.^101^

The suggestion throughout has been that Krafft-Ebing’s attempt to coerce homosexuality, androgyny, and identification of sex-of-self into a single aetiological framework took time to develop, and was on the whole hardly original. Rather, the 1880s nosological and aetiological responses to Westphal rendered psychological sex an even more layered medical palimpsest than it had already been for centuries. As argued, Krafft-Ebing came in at the tail end of centuries of modern takes on the ‘Scythian malady’, which had started out somatic and occasionally neurophysiological but had long been gravitating toward exercises in historico-environmental neuropsychiatry, well before nineteenth-century alienists began to claim a professional identity. By the 1880s, it had been conceptualised for over a century in terms of feminisation, *pathicism* (sexual passivity understood as a condition), and intellectual monomania. The Index-catalogue of the Library of the Surgeon-General’s Office had the neologistic, undefined heading of *morbus Scythicus* from 1888, featuring a bibliography that was considerably more complete than Krafft-Ebing’s.^102^ Although seemingly only a fraction came to his attention, Krafft-Ebing’s citations to this ancient problem can be seen multiplying by the year, refracting it quite differently during all stages of his thinking. In his 1879 psychiatry textbook, he had only briefly and anachronistically characterised the Scythian malady as an example of ancient psychiatry, specifically of ‘epidemic mental confusion’ (*epidemische Geistesverwirrung*). This unoriginal call echoed a consensus between authors ranging from Morel to Von Feuchtersleben and Maximilian Leidesdorf (1816–1889), the last of whom Krafft-Ebing later succeeded as chair of the psychiatric clinic of the *Niederösterreichische Landesirrenanstalt.* ^103^ The reference and qualification were retained as late as Kraft-Ebing’s textbook’s third (1888) edition but removed from the fourth (1890): Krafft-Ebing’s nosology had been refined, superseding his medical history.^104^ The ancient ‘feminine disease’ had been absent from the first, 1886, edition of Krafft-Ebing’s *Psychopathia sexualis*, and its rethinking appears substantively inspired by discussions at the American Neurological Association. As shown in both his citations and his nosology, only in 1889 did Krafft-Ebing see clear cause to dig up and read additional literature on the Scythian malady, clearly via Hammond’s 1882 article, from which he gleaned and traced multiple earlier sources. The question only now became central to Krafft-Ebing’s clinical thought and re-reading of case material: which cases of gender-crossing did, and which cases did not, allow a psychosis verdict? Krafft-Ebing seemed to have required a period of build-up of courage in his decision-making at this point: while in *Psychopathia*’s fourth edition the matter still only seemed likely, in the fifth edition he definitively interpreted the Scythian *anarieîs* as evidencing ‘superstitious interpretation of *effeminatio*, as already indicated by Hippocrates’ – unlike more ‘progressive’ cases of true delusion (‘paranoid sexual transformation’) where culture did not support such interpretations. Hammond’s two-spirit *mujerados*, too, did not warrant the latter reading.^105^ Where Krafft-Ebing’s initial consideration had been Hippocratically epidemiological (psychosis was rare, historical and *endemic* psychosis rarer), in 1890 Krafft-Ebing appears to simply have agreed with Hammond’s 1882 ethno-psychological opinion, rendering gender-transing a uniquely Western problem of sexual degeneration.

Having discussed the Scythian malady since 1891, Albert Moll weighed what he gradually found out were multiple accounts of it, and refrained from pinning himself down, although in 1899 he did maintain, as had other authors including aforementioned Emminghaus, that, ‘of course, contrary sexual sensation must be completely separated from mental disorders in which the insane man considers himself a woman [*sich … für ein Weib hält*] and consequently dresses accordingly and wants to associate with men’.^106^ That the Scythian disease was ‘an affection consisting in the performance of homosexual acts has been made probable by [Julius] Rosenbaum’, he wrote in 1893 and still in 1899. So, even preeminent sexologist Moll at this late point still had little interest in questioning and probing the cross-cultural and transhistorical nuances between cross-gender identification and cross-sex delusion. But it was in this undertheorized sense that Moll, among many others, as seen, clearly *did* beat Hirschfeld to the explicit separation between cross-‘sex’ identification and same-sex sexual inclination. American authors, in comparison, were on the whole less discerning still, despite 1890s translations of Krafft-Ebing and Schrenck-Notzing, and Havelock Ellis’ *Sexual Inversion.* First published in 1906, Joseph Parke’s American sexuality textbook still elaborately discussed Krafft-Ebing’s notions of ‘delusional eviration’ and ‘delusional masculinity’.^107^ Parke claimed to know of two such ‘delusional’ cases. At the same time, in an attempt to conform to 1890s Anglophone sexology, he arranged both the *anarieîs* and *mujerados* under a separate makeshift ethnopsychiatric heading: ‘religio-mystical inversion’.^108^ Even Iwan Bloch initially grouped them under the ethnological heading of religious ‘pederastic effemination’.^109^

Understanding clinical nuance and relevance at home long proved challenging. Moll’s reading in 1891, retained in 1893, was the following: ‘Perhaps we can think of the matter in a similar way to delusions and obsessions [*Wahn- und Zwangsvorstellungen*]; while the latter is an awareness of the disease or at least the desire to suppress the pressing ideas, in the case of delusions these are no longer recognized as pathological.’^110^ These considerations were not trivial: where male same-sex sex seemed in play, ‘paranoia’ spoke to vital legal dimensions of the *Urningsfrage.* As one American doctor proposed in 1897, though not without inviting discussion, ‘If there is here a true delusion of sexual transformation, it is equivalent to a monomania and the subject should receive asylum treatment’.^111^ And as one author had noted already in 1882:

among the 17 cases [of sexual inversion] so far recorded in the literature and carefully studied, there is none in which the anomaly occurs in mente sana. […] It should be avoided that out of badly applied moral indignation the paragraph of the law [in Austria then prohibiting ‘lewdness against the order of nature’, male and female] is used against unfortunate degenerates whose libertas judicii et consilii [ie., the faculty of discrimination and freedom of intention/deliberation/consideration] is in question by the ‘conflict of nature’ of their self-awareness [den „Zwiespalt der Natur” ihrer Selbstempfindung].^112^

In other words, at least *some* indicted inverts could prove to be not only comorbidly psychotic (and this possibly in nontrivial numbers) but psychotics *by clinical definition.* The at least theoretical problem was interestingly left unsolved by Krafft-Ebing, who, in 1901, rolled back the blanket psychiatric verdict on *Conträrsexuale.* The ‘people who believe they belong to the opposite sex’ he had left in a legal limbo, as others later observed.^113^

Barring much explicit reflection on this medicolegal point, actual cases of *metamorphosis sexualis paranoica* remained rare until Hirschfeld’s *Die Transvestiten.* Eulenburg illustratively agreed that such a condition constituted an ‘end-stage of sexual inversion’ (*Endstufe psychosexualer Inversion*), whereas bisexuality was a preliminary stage (*Vorstufe*).^114^ In 1895, he offered a brief but purportedly typical example of his own. Here was an intelligent and otherwise lucid thirty-seven-year-old man who ‘because of certain physical changes observed over the past 4-5 months’ relating to secondary sexual characteristics ‘had come to the “firm conviction” that he “was a 37-year-old man, husband and father of a son, who will become a woman”’. A letter revealed ‘he’ had been reading, and thus must have been ‘apparently significantly influenced’ by, a passage on the feminising effects of industrialisation in Ellis’s *Man and Woman* (1894), which had appeared in German translation that same year. It seems that Eulenburg was suggesting we understand this account of distressing, purportedly recent, sudden, and even paroxysmal feminisation as self-deluding intellectualisation of a pre-existent, though disavowed because shameful, androgyny. If we presume the distressing symptom could have been gynecomastia, any condition sufficiently altering the oestrogen/androgen ratio could have explained the symptoms. Regardless, *sex-paranoia* remained a more layered, more psychological, and more diverse set of conditions than could be appreciated from unsolicited letters. Their relation to ‘sexual inversion’ remained a concomitantly qualified one.

Hirschfeld’s circle, too, only slowly gravitated to an appreciation of gender crossing as a differential-diagnostic problem. In 1902, Eugen Wilhelm, the *Wissenschaftlich-humanitäres Komitee*’s legal expert, found occasion to explicate, inaccurately, that ‘Of course, no one has thought of throwing the contrary sexual sensation together with delusional ideas’.^115^ However: ‘Contrary-sexuals [*Konträre*] would suffer from delusional ideas if, for example, he took another man to be a woman or himself to be a woman, if, for example, contrary-sexual [i.e. homosexual] sensations and the sex change delusion [*Wahn der Geschlechtsverwandlung*] were identical’. The spectrum of *Konträrsexualismus* clearly did not include transgender identification or gender dysphoria, or for some other reason, warrant more ‘clinical’ nuance, at this point.

Hirschfeld later agreed with the existence of Krafft-Ebing’s ‘paranoia’ cases, of which Hirschfeld himself claimed to know two. Unlike *transvestites*, these people had delusions about their primary and secondary sexual characteristics, their real sex (*wahres Geschlecht*).^116^ Transvestitism was defined in contrast with such cases: a successful, if melancholic, grappling with the reality that was sex, specifically the historical fact that was natal sex, requiring a mild, self-pleasing self-deception that one could pass ‘entirely’ – as had one been born that way. *Transvestitismus*, at least in this 1910 formulation, included, though it was not defined by one’s relating to the unrealistic *idea*, the unreal, unattainable scenario, of fully embodying another sex, entertained but never fully believed.

Sexual nonconformity and sexual realism, in summary, share a long and eventful medical history. In Krafft-Ebing’s casuistics from 1888 onward, both delusions (*Wahnen*) and hallucinations related to genitalia and masturbation became confounded by a new nuance of identity – the lasting awareness of self. Some of Krafft-Ebing’s early pertinent cases, especially the earliest, case 51 of 1888, would in a later timeframe have risked being diagnosed with what Eugen Bleuler, in 1908, came to call *schizophrenia.* In 1911, Bleuler himself paid lip service to ‘delusional ideations of sexual transformation’ (*Wahnideen der Geschlechtstransformation*), an unreferenced and by now woefully undertheorized expression lacking case studies.^117^ The question of ‘doubt about sexual identity’ in male schizophrenics would remain a distinct clinical problem, eventually alongside the emergent concept of ‘gender identity’.^118^ Psychodynamic theorising provided a dual backdrop. Where in 1953 Manfred Bleuler opined that his father Eugen would have agreed that ‘schizophrenics are almost invariably, if not indeed invariably, in doubt about the sex to which they belong’, this seems to echo grand psychodynamic hypotheses, such as that ‘in man every case of emotional neurosis or psychosis is the result of more or less conflict and confusion involving bisexual differentiation.’^119^ During the 1920s, ‘delusions’ were indeed repeatedly considered material for psychodynamic and symbolic readings.^120^ Various early twentieth-century case studies of cross-dressing reported (pre-existing) delusions that are hard to reduce to identity issues. Such cases stood more of a chance to pique the interest of the law and of the clinical case reporter, certainly when considered to require hospitalisation. In any case, from the late 1880s, the onset, precise content, and course of atypical sexual ideations mattered, and these factors have continued to matter ever since. As explored to some extent by Rainer Herrn, Hirschfeld himself continued to wrestle with this problem of apparent heterogeneity well into the 1920s, and provided various solutions to subclassification, all little successful. Sex, by now, had of course proven less and less fixed. From 1910, the once far-out ideas of social and medical transitioning became increasingly thinkable, with various legal, opotherapeutic, cosmetic, and surgical avenues explored in Berlin.^121^ What was real sex, and what realistic sexually, were proving changeable constructs. So was ‘the idea of having changed sex’. If we conceptualise psychosis as a discursive wedge between self (one’s inner dialogue) and non-self (e.g. voices inside one’s head), there is, naturally, considerable scope to historicize it in gendered terms of both aetiology and symptomatology.^122^ As with *hysteria* and with *sexual trauma*, cultural-historical factors weigh in heavily on definition, causality, and presentation. Psychosis can be considered a gradual phenomenon for which most people, fully regardless of gender identity, have some degree of susceptibility, chronic exclusion and humiliation being potent risk factors. Furthermore, as with reasonably effective hormonal treatments, reasonably effective antipsychotic options in pharmacology did not become available until the 1930s.

## Footnotes

1

Joseph Jörger , ‘Das inducirte Irresein’, Allgemeine Zeitschrift für Psychiatrie und psychisch-gerichtliche Medicin 45, no. 4 (1889), 307–63, esp. 322–53, co-published as a doctoral thesis to the University of Basel (*Das inducirte Irresein.* Berlin: n.p., 1888), here especially 16ff. Richard von Krafft-Ebing was the first co-editor of the cited journal but never referenced the cited article.

2
*Ibid.*, 350.

3

Geertje Mak , Doubting Sex: Inscriptions, Bodies and Selves in Nineteenth-Century Hermaphrodite Case Histories (Manchester: Manchester University Press, 2013), 186 ; Rainer Herrn, ‘Das Geschlecht Ruht Nicht im Körper, Sondern in der Seele: Magnus Hirschfeld’s Strategien bei Hermaphroditengutachten’, in Neue Gesellschaft für Bildende Kunst (ed.), *0-1 intersex. Das Zweigeschlechter-System als Menschenrechtsverletzung* (Berlin, 2005), 55–71.

4 Mak, *op.cit.* (note 3), 209.

5
*Ibid.*, 230; Sabine Mehlmann , ‘Sexualität und Geschlechtlichkeit vom Geschlechtscharakter zur Geschlechtsidentität’, in Ursula Ferdinand , Andreas Pretzel and Andreas Seeck (eds.), Verqueere Wissenschaft? (Münster: LIT, 1998), 35–50 ; Simon Joyce , LGBT Victorians: Sexuality and Gender in the Nineteenth-Century Archives (Oxford: Oxford University Press, 2022), 71ff.

6 Mak, *op.cit.* (note 3), 231.

7

Geertje Mak , ‘Magnus Hirschfeld’s 1899 psychobiological questionnaire: The paradoxes of de-narrativizing sexual and gender nonconformity’, Intellectual History Review 32, no. 3 (2022), 599–617 , 605.

8

Jules Gill-Peterson , Histories of the Transgender Child (Minneapolis: University of Minnesota Press, 2018), 100 , 116.

9

Diederik Janssen , ‘Melancholia Scytharum: the early modern psychiatry of transgender identification’, History of Psychiatry 32, no. 3 (2021): 270–8833855893 10.1177/0957154X211006253PMC8339897.

10 (Jean Le Rond) d’Alembert, *Éloges lus dans les séances publiques de l’Académie* française (Paris: Panckoucke & Moutard, 1779), 315.

11 (Jean Le Rond) d’Alembert, *Histoire des membres de l’académie françoise, morts depuis 1700 jusqu’en 1771* […]. Vol. 4 (Paris: Moutard, 1786), 309.

12 AR, [Book review, Nov. 1742: *La Vie de Monsieur l’abbé de Choisy*]. *Nouvelle bibliothèque ou histoire littéraire des principaux écrits qui se publient, Octobre 1738*–*Juin 1744* (The Hague: Pierre Paupie, 1742), 425–7, here 426.

13 L…, née de L. P…, Caroline (Christophe-Paulin de La Poix de Fréminville), *Essai sur l’influence physique et morale du costume féminin* (Paris: Imprimerie de Stahl, 1831), 7.

14
*Ibid.*, 6, 7.

15
*Ibid.*, 5, 6, 10.

16 Gustave Macé, *Gibier de Saint-Lazare* (Paris: G. Charpentier & co., 1888), 227.

17 See Ross Brooks , ‘Transforming Sexuality: The Medical Sources of Karl Heinrich Ulrichs (1825–95) and the Origins of the Theory of Bisexuality’, Journal of the History of Medicine and Allied Sciences 67, no. 2 (2012), 177–216 21081540 10.1093/jhmas/jrq064.

18 General accounts include Sabine Mehlmann , *Unzuverlässige Körper*. Zur Diskursgeschichte des Konzepts geschlechtlicher Identität (Königstein im Taunus: Ulrike Helmer Verlag, 2006), 164ff; Julien E. Fischer, *The Lure of Origins: Sexology and the Trans Autobiographical Mandate* (Ph.D. dissertation, Duke University, 2023), ch. 1.

19

Ira B. Pauly , ‘Male psychosexual inversion: Transsexualism: A review of 100 cases’, Archives of General Psychiatry 13, no. 2 (1965): 172–81, 17914314763 10.1001/archpsyc.1965.01730020074009.

20

Richard von Krafft-Ebing , ‘Ueber sexuelle Perversionen’, in E. von Leyden & F. Klemperer (eds.), Die Deutsche Klinik am Eingang des 20. Jahrhunderts in akademischen Vorlesungen, Vol. 6. (Berlin: Urban & Schwarzenberg (1901/1906), 113–54, 115; Krafft-Ebing, *Psychopathia sexualis* […]. 12th ed. (Stuttgart: Ferdinand Enke, 1903), 35.

21 See, for example, the rare and few theoretical comments on the ‘Entwickelung des Geschlechtsbewusstseins’, in J. Oldřich Kramář, *Die Hypothese der Seele: ihre, Begründung und metaphysische Bedeutung.* 2 vols. (Leipzig: Duncker & Humblot, 1898), 1:375–6.

22 E. (Eugène) Gley, ‘Les Aberrations de l’instinct sexuel: D’après des travaux récents’, *Revue philosophique de la France et de l’étranger* 17 (1884), 66–92, 68.

23

E. Toulouse , [Book review]. Lancette française: Gazette des hôpitaux civils et militaires 66, no. 34 (1893), 329–30, 330.

24 ‘l’assurdità dell’idea fondamentale dell’io’: Arrigo Tamassia, ‘Sull’inversione dell’istinto sessuale’, *Rivista sperimentale di freniatria e di medicina legale* 4 (1878), 97–117, 116.

25 (Ernst Joseph Gustav) de Valenti, *Medicina clerica, oder Handbuch der Pastoral-Medizin für Seelsorger, Pädagogen und Aerzte: nebst einer Diätetik für Geistliche.* Vol. 2, *Die specielle Pastoral-Medizin* (Leipzig: K.F. Köhler, 1832), esp. 51ff, 324–5.

26
*Ibid.*, 73.

27 A. (Alexandre-Jacques-François) Brierre de Boismont, ‘Aliénation mentale’, in (Jean-Baptiste) Glaire and vicomte (Joseph Alexis) Walsh (ed.), *Encyclopédie catholique*, (Paris: Parent-Desbarres, 1840), 2:35–48, 45.

28 A. (Achille-Louis) Foville, ‘Aliénation mentale’, In *Dictionnaire de médecine et de chirurgie pratiques*, Vol. 1 (Paris: Gabon [etc.], 1829), 484–586, 498.

29

F.-J. Gall , Sur les fonctions du cerveau et sur celles de chacune de ses parties, Vol. 3 (Paris: Author & Boucher, 1823), 122 .

30 François-Joseph-Victor Broussais, *De l’irritation et de la folie* […] (Paris: Delaunay, 1828), 376.

31 Wilhelm Griesinger, *Die Pathologie und Therapie der psychischen Krankheiten* (Stuttgart: Adolph Krabbe, 1845), 184, referring to (François) Lallemand, *Des Pertes séminales involontaires* (Paris: Bechet jr., 1836), 64–6. The same opinion as Griesinger’s is recorded by Johann August Schilling, *Psychiatrische Briefe oder die Irren, das Irresein und das Irrenhaus.* 2^nd^ ed. (Augsburg: Schlosser, 1866), 148–9.

32 Max[imien] Parchappe, ‘Symptomatologie de la folie [cont’d]’, *Annales médico-psychologiques* (Ser. 2) 3 (1851), 40–97, 54.

33 B[enjamin] Ball, *Leçons sur les maladies mentales* (Paris: Asselin & co., ‘1880-3’), 170–1. Ball elsewhere again distinguished what the Germans called *conträre Sexualempfindung* as homosexual attraction with aversion to opposite sex (rubrified under *idées érotiques*), and the fact that ‘some patients imagine that they have changed sex’ (rubrified under *idées de transformation corporelle*). B[enjamin] Ball and Ant[oine] Ritti, ‘Délire’, In *Dictionnaire encyclopédique des sciences médicales* (Paris: G. Masson & son, P. Asselin, 1882), 315–408, 395.

34

Heinrich Neumann , Lehrbuch der Psychiatrie (Erlangen: Ferdinand Enke, 1859), 67 .

35

de Montyel, ‘De la maladie des scythes’, Annales médico-psychologiques (ser. 5) 17 (1877), 161–74; William Hammond , ‘The disease of the Scythians (morbus feminarum) and certain analogous conditions’, Journal of Neurology and Psychiatry 1, no. 3 (1882) 339–55, 353.

36 Antigono Raggi, ‘Aberrazione del sentimento sessuale in un maniaco ginecomasta’, *Annali universali di medicina e chirurgia* [Milan] 259 (April, 1882), 289–98; *Reale di Scienze e Lettere Rendiconti Istituto Lombardo* (Ser. 2) 15 (1882), 177–80. The case was picked up by Tarnowski but not by Krafft-Ebing.

37 A.C.M., ‘Enareans’, *Notes & Queries* IX, no. 223 (1854), 101–3, 102.

38 [Adolf] B[astian], [Untitled]. *Zeitschrift für Ethnologie* 1 [1869], 88–9.

39 Adolf Bastian, *Beiträge zur vergleichenden Psychologie: Die Seele und ihre Erscheinungsweisen in der Ethnographie* (Berlin: Ferd. Dümmler, 1868), 235ff.

40

Adolf Bastian , *Der Mensch in der Geschichte: Zur Begründung einer psychologischen Weltanschauung* . Vol. 2: Psychologie und Mythologie (Leipzig: Otto Wigand, 1860), 32 ; quoting Johann Spielmann , Diagnostik der Geisteskrankheiten: für Ärzte und Richter (Vienna: Braumüller, 1855), 404–5.

41 C[arl] Westphal, ‘Die conträre Sexualempfindung: Symptom eines neuropathischen (psychopathischen) Zustandes’, *Archiv für Psychiatrie und Nervenkrankheiten* 2, no. 1 (1869), 73–108, 102–3.

42 [Auguste] Chatelain, in *Annales médico-psychologiques* (Ser. 5) 6 (1871), 285–8. This is quoted at length in Joseph Tissot, *La Folie considérée surtout dans ses rapports avec la psychologie normale* […] (Paris: A. Marescq sr., 1877), 213.

43 Konrad Alt, ‘Das Symptom der Personenverwechslung bei Geisteskranken’, *Allgemeine Zeitschrift für Psychiatrie und psychisch-gerichtliche Medicin* 44 (1888), 50–77, 71, cf. 51.

44 ‘Therianthropie ähnliche pathologische Erscheinung’, a neologism, from GR θηρίον ‘wild animal’: W.H. (Wilhelm Heinrich) Roscher, *Das von der „Kynanthropie” handelnde Fragment des Marcellus von Side* (Leipzig: S. Hirzel, 1896), 17n42^b^, cf. 25n61.

45

Ernest Martin , Histoire des monstres depuis l’antiquité jusqu’à nos jours (Paris: C. Reinwald & co., 1880), 324–9.

46
*Ibid.*, 325.

47

Sylvie Chaperon , Les Origines de la sexologie, 1850–1900 (Paris: éditions Louis Audibert, 2007).

48 Representative works include those by Ribot and, e.g. Charles Richet , L’homme et l’intelligence: fragments de physiologie et de psychologie (Paris: Alcan, 1884); Alexandre Herzen , Le Cerveau et l’activité cérébrale au point de vue psycho-physiologique (Paris: J.-B. Baillière & son, 1887), see 275ff; Henri Bourru and Ferdinand Burot , Variations de la personnalité (Paris: J.-B. Baillière & son, 1888); Louis-Émile Dumas , Transformations de la personnalité; erreurs de personnes chez les aliénés. Doctoral thesis, Université de Bordeaux (Paris: Rougier, 1886); Alfred Binet , Les Altérations de la personnalité (Paris: Félix Alcan, 1892); William T. Preyer , Die Seele des Kindes (Leipzig: Th. Grieben, 1882), 359ff.

49 William James, *The Principles of Psychology.* 2 vols. (New York: Henry Holt & co., 1890), 1:291, 292, ital. in orig., also 400, cf. Magnus Hirschfeld, *Die Transvestiten* (Berlin: Alfred Pulvermacher & co., 1910), 257ff.

50 See Giovanni Pietro Lombardo and Renato Foschi , ‘The concept of personality in 19th-century French and 20th-century American psychology’, History of Psychology 6, no. 2 (2003), 123–4212817602 10.1037/1093-4510.6.2.123.

51 Th[éodule-Armand] Ribot, ‘Les Bases affectives de la personnalité’, *Revue philosophique de la France et de l’étranger* 18 (1884), 138–72, esp. 153–7. This passage was notably spotted, all too briefly, as ‘mentioning contrary sexual feeling’ by Eugen Wilhelm in the *Jahrbuch für sexuelle Zwischenstufen* 2 (1900), 431.

52 ‘Some obscure organic disorder occasionally so modifies the cœnæsthesis [i.e. organic ego-sensibility, sense of bodily unity] that the subject believes himself to be made of stone, or butter, or wax, or wood; to have changed sex [an English translation gives: *that he is of the opposite sex*], or that he is dead’, Th[éodule-Armand] Ribot, ‘Les Désordres généraux de la mémoire’, *Revue philosophique de la France et de l’étranger* 10 (1880), 181–214, 202.

53

Henry Maudsley , Body and Mind: An Inquiry into Their Connection and Mutual Influence to Mental Disorders (London: MacMillan & Co., 1870), 32 .

54
*Ibid.*

55
*Ibid.*, 77–8.

56 Ribot, *op.cit.* (note 51), 156; Richard von Krafft-Ebing, *Psychopathia sexualis. Eine klinisch-forensische Studie* (Stuttgart: Ferdinand Enke, 1886), 15, 33; Marie, ‘Eunuchisme et psychologie sexuelle’, *Journal de psychologie normale et pathologique* 4 (1907), 113–27.

57 Ribot, *op.cit.* (note 51),157.

58

Evgenīĭ Pelikan , Gerichtlich-medicinische Untersuchungen über das Skopzenthum in Russland, nebst historischen Notizen. Trans. Nicolaus Iwanoff (Giessen: J. Ricker, 1876) , see 104–36.

59 A[ugust] Cramer, ‘Die conträre Sexualempfindung in ihren Beziehungen zum § 175 des Strafgesetzbuches’, *Berliner klinische Wochenschrift* 34, no. 43 (1897), 934–6, 936.

60

Richard von Krafft-Ebing , Neue Forschungen auf dem Gebiet der Psychopathia sexualis. 2^nd^ ed. (Stuttgart: Ferdinand Enke, 1891), 94–5.

61

Rudolf Arndt , Lehrbuch der Psychiatrie für Ärzte und Studirende (Berlin/Vienna: Urban & Schwarzenberg, 1883), 142–3, 150, 172–3.

62
*Ibid.*, 173.

63
*Ibid.*, 142, 143.

64 Krafft-Ebing, *op.cit.* (note 56), 25, 34.

65 Krafft-Ebing, *op.cit.* (note 60), 78ff.

66 Fischer, *op.cit.* (note 18).

67 [Léon] De Rode, [Review: *Neue Forschungen*]. *Bulletin de la Société de médecine mentale de Belgique* (for 1891) 217–21, 220.

68
*Mania metamorphosis* appears in Johann Christian Reil’s *Rhapsodieen über die Anwendung der psychischen Curmethode auf Geisteszerrüttungen* (Halle: Curt, 1803), 337–8, inspired by Chiarugi’s 1794 term, *melancholia metamorphosis*, the latter referring to Thomas Willis’ *De anima brutorum.*

69 Kühn, [Book review], *Zeitschrift für Medizinalbeamte* 6, no. 16 (1893), 409–11, 410.

70 Westphal, *op.cit.* (note 41), 98, 107n, cf. ‘Gradunterschiede’, 105.

71

Heinrich Schüle , Handbuch der Geisteskrankheiten (Leipzig: F.C.W. Vogel, 1878), 117 .

72
*Ibid.*, 115.

73 [Richard] v[on] Krafft-Ebing, ‘Psychopathologie’, *Zeitschrift für die gesamte Strafrechtswissenschaft* 2 (1882), 366ff, 373–5; Hermann v[on] Voigt, ‘Die Psychiatrie des Jahres 1881’, *Jahrbuch der praktischen Medizin* (year 1882), 197ff, 224–5.

74 ‘Sexual impulses also appear at this stage in the form of contrary sexual sensations; this is where women disguised as men belong and vice versa, often with the most disgusting cynicism of demoralized prostitutes’, Schüle, *op.cit.* (note 71), 420–1.

75

Hermann Emminghaus , Allgemeine Psychopathologie zur Einführung in das Studium der Geistesstörungen (Leipzig: F.C.W. Vogel, 1878), 53–4. With reference to Leuret and Lallemand, Griesinger had earlier classified instances of sex-crossing ‘belief’ or ‘delusion’ (Wahn der Geschlechtsumänderung) under *melancholia metamorphosis*: *Die Pathologie und Therapie der psychischen Krankheiten, für Aerzte und Studirende* (Stuttgart: Adolph Krabbe, 1845), 184–5.

76 ‘Sitzung den 5. Februar 1876’ [discussing (Hermann) Emminghaus, ‘Ueber einige ethnologische Aequivalente des Irreseins’], *Sitzungsberichte der physikalisch-medicinischen Gesellschaft in Würzburg für das Gesellschaftsjahr 1876*, IVff, see VI. The text seemingly remained unpublished.

77

Arnold Pick , ‘Die psychiatrischen Anschauungen La Mettrie’s (1709–1751)’, Jahrbücher für Psychiatrie 1 (1879), 52–7, 56. In 1883 Pick would notably go on to review Hammond’s important 1882 article (*op. cit.*, note 35), for the *Neurologisches Centralblatt.*

78 B. (Veniamin Mikhaĭlovich) Tarnovskiĭ, *Die krankhaften Erscheinungen des Geschlechtssinnes: eine forensisch-psychiatrische Studie* (Berlin: August: Hirschwald, 1886), 15, 16.

79

Richard von Krafft-Ebing , ‘Zur ‘conträren Sexualempfindung’ in klinisch-forensischer Hinsicht’, Allgemeine Zeitschrift für Psychiatrie und psychisch-gerichtliche Medicin 38 (1882), 211–27, 221. By 1882, cross-dressing was clearly part of the ‘inversion’ syndrome though neither this nor gender identity was as yet tied to this admittance of developmental stages (Entwicklungsstufen): ‘Not infrequently there is a true urge to dress, adopt a haircut, shave, etc., according to the individual’s felt gender role [geschlechtlichen Rolle in welcher sich das Individuum fühlt]’ (220). Of note is that in 1882 Krafft-Ebing, next to his own 1877 article, anachronistically invokes authorities such as Julius Rosenbaum and Ambroise Tardieu as sources pertinent to *conträre Sexualempfindung* (211n2) but duly omits these references in a 1883 text.

80 M[üller], ‘Original-Vereinsberichte’, *Centralblatt für Nervenheilkunde, Psychiatrie und gerichtliche Psychopathologie* 10, no. 8 (April 15, 1887), 229–31.

81 Richard von Krafft-Ebing, *Psychopathia sexualis* […]. 2nd ed. (Stuttgart: Ferdinand Enke, 1887), 99.

82 Richard von Krafft-Ebing, *Psychopathia sexualis* […]. 3rd ed. (Stuttgart: Ferdinand Enke, 1888), 129.

83 Emil Kraepelin, *Compendium der Psychiatrie* […] (Leipzig: Ambroise Abel, 1883), 284.

84

Richard von Krafft-Ebing , Lehrbuch der Psychiatrie auf klinischer Grundlage für praktische Ärzte und Studirende. 3^rd^ ed. (Stuttgart: Ferdinand Enke, 1888), 433–7; cf. 4^th^ ed. (Stuttgart: Ferdinand Enke, 1890), 430ff. For its history see the international overview touching upon the problem of identity/identification disorders (‘perversion de la personnalité’) by J[ules] Séglas, ‘La Paranoia délires systématisés et dégénerescences mentales. Historique et critique’, *Archives de neurologie* 13 (1887), 62–76, 221–32+393–406.

85 R[ichard] von Krafft-Ebing, *Lehrbuch der Psychiatrie auf klinischer Grundlage für practische Ärzte und Studirende.* 3 vols. (Stuttgart: Ferdinand Enke, 1879), 2:73–6.

86

Richard von Krafft-Ebing , Die Sinnesdelirien: ein Versuch ihrer physio-psychologischen Begründung und klinischen Darstellung (Erlangen: Ferdinand Enke, 1864), 41 , 42–3. See especially the advance between Krafft-Ebing’s ‘Irresein durch Onanie bei Männern’, *Allgemeine Zeitschrift für Psychiatrie und psychisch-gerichtliche Medicin* 31, no. 4 (1878), 425–40, and his ‘Ueber primare Verrücktheit auf masturbatorischer Grundlage bei Männern’, *Der Irrenfreund* 20, nos. 9/10 (1878), 129–46; ‘Ueber Irresein im Klimacterium’, *Allgemeine Zeitschrift für Psychiatrie und psychisch-gerichtliche Medicin* 34, no. 4 (1878), 407–17; and ‘Untersuchungen über Irresein zur Zeit der Menstruation. Ein klinischer Beitrag zur Lehre vom periodischen Irresein’, *Archiv für Psychiatrie und Nervenkrankheiten* 8 (1878), 65–107.

87 Kraepelin, *op. cit.* (note 83), 300.

88 Krafft-Ebing, ‘Ueber Irresein’ (note 86), 407, 408.

89 H[enri] Dagonet with others, *Traité des maladies mentales* (Paris: J.-B. Baillière & son, 1894), 85.

90 R. (Rudolf) Leubuscher, *Grundzüge zur Pathologie der psychischen Krankheiten. Erläutert an Krankengeschichten* (Berlin: G. Reimer, 1848), 74ff; J.L.C. (Jacobus Ludovicus Conradus) Schroeder van der Kolk, *Handboek van de pathologie en therapie der krankzinnigheid* (Utrecht: Van der Post, 1863), 217–34.

91

P.F. Spaink , ‘Bijdrage tot de casuistiek der Urningen’, Psychiatrische Bladen 11 (1893), 143–65, esp. 144–6.

92 Krafft-Ebing, *op.cit.* (note 60), 71. The chapter picks up from three case studies (two autobiographic accounts) of ‘effeminatio’ in the first edition of 1890 (here 62ff).

93
*Ibid.*

94 Krafft-Ebing never cited Ribot’s account (nor Ernest Martin’s). Some reflection on same-sex sociality in children, apropos the notion of sexual inversion, is found in Martin Cohn, ‘Ueber die Psychosen im kindlichen Alter [cont’d]’, *Archiv für Kinderheilkunde* 4 (1883), 101–7, 104.

95 Richard von Krafft-Ebing, *Psychopathia sexualis* […]. 4th ed. (Stuttgart: Ferdinand Enke, 1889), 85.

96 Richard von Krafft-Ebing, *Psychopathia sexualis* […]. 5th ed. (Stuttgart: Ferdinand Enke, 1890), 92–5.

97 C[olonel] B. Burr, ‘Paranoia, with delusions of change in sex. Psychological observations and surgical notes’, *Buffalo Medical and Surgical Journal* 33, no. 4 (1893), 193–7. The surgeon (Walter Porter Manton, 1858–1925) also mentioned the case: ‘What results may be expected from celiotomy in insanity, hystero-epilepsy and kindred neuroses? Remarks with illustrative cases’, *Physician and Surgeon* 16, no. 4 (1894), 145–55, 149.

98 Burr, *op.cit.* (note 97), 196.

99 Leopold Löwenfeld, *Sexualleben und Nervenleiden: Die Nervösen Störungen sexuellen Ursprungs* […]. 3rd ed. (Wiesbaden: J. F. Bergmann, 1903), 243–4n1.

100 Roller (reported by Bruns), ‘Casuistische Mittheilungen’, *Neurologisches Centralblatt* 10, no. 11 (June, 1891), 350–1.

101 ‘Versammlung des Vereins der Irrenärzte Niedersachsens und Westfalen zu Hannover am 1. Mai 1891’, *Allgemeine Zeitschrift für Psychiatrie und psychisch-gerichtliche Medicin* 48, no. 4 (1892), 405ff, esp. 420–4, 423.

102
*Index-catalogue of the Library of the Surgeon-General’s Office.* Vol. 9 (Washington, DC: Government Printing Office, 1888), 445, and Ser. 3, Vol. 11 (Washington, DC: Government Printing Office, 1906), 79.

103 Krafft-Ebing, *op.cit.* (note 85), 1:15; Ernst Freiherr von Feuchtersleben, *Lehrbuch der ärztlichen Seelenkunde* (Vienna: Carl Gerold, 1845), 22; Max Leidesdorf, *Lehrbuch der psychischen Krankheiten.* 2^nd^ ed. (Erlangen: Ferdinand Enke, 1865), 2.

104 Krafft-Ebing, *op.cit.* (note 84), 40.

105 Krafft-Ebing, *op.cit.* (note 95), 82–3. Charles Gilbert Chaddock, notably added a long footnote (202n) to the section on ‘Eviration and Defemination’ discussing Holder’s 1889 discussion of the Native-American two-spirit *Bote*; this is not found in the later Rebman and Klaf translations.

106

Albert Moll , Die konträre Sexualempfindung; Mit Benutzung amtlichen Materials. 3rd ed. (Berlin: Fischer, 1899), 425–6.

107 J[oseph] Richardson Parke, *Human Sexuality* (Philadelphia: Professional Publishing Co., 1906/1912), 263ff, 295–6, 323.

108
*Ibid.*, 323.

109 Iwan Bloch, *Beiträge zur Aetiologie der Psychopathia sexualis.* Vol. 1 (Dresden: H.R. Dohrn, 1902), 122n4.

110 Albert Moll, *Die conträre Sexualempfindung; Mit Benutzung amtlichen Materials* (Berlin: Fischer, 1891), 199n1.

111

C.W. Allen , ‘Report of a case of psycho-sexual hermaphroditism’, Medical Record 51 (May 8, 1897), 653–5, 655.

112 [Heinrich] Sterz, ‘Beitrag zur Lehre von der “conträren Sexualempfindung”’, *Jahrbücher für Psychiatrie* 3 (1882), 221–8, 222.

113

Heinrich Gräf , ‘Über die gerichtsärztliche Beurteilung perverser Geschlechtstriebe’, Archiv für Kriminal-Anthropologie und Kriminalistik 34 (1909), 45–122, 67.

114 A[lbert] Eulenburg, ‘Neuropathia sexualis virorum’, In *Klinisches Handbuch der Harn- und Sexualorgane.* Vol. 4. Ed. A[lbert] Eulenburg *et al.* (Leipzig: F.C.W. Vogel, 1894), 1–79, 69.

115
*Jahrbuch für sexuelle Zwischenstufen* 4 (1902), 735.

116 Hirschfeld, *op.cit.* (note 49), 235.

117

Eugen Bleuler , Dementia praecox, oder, Gruppe der Schizophrenien (Leipzig/Vienna: Franz Deuticke, 1911), 101 , 118.

118 E.g. N. L. Gittleson and S. Levine , ‘Subjective ideas of sexual change in male schizophrenics’, British Journal of Psychiatry 112, no. 489 (1966), 779–8210.1192/bjp.112.489.7795966551.

119 Cit. in *ibid.*, 779; Edward J. Kempf, ‘Bisexual Factors in Curable Schizophrenia’, *Journal of Abnormal and Social Psychology* 44, no. 3 (1949), 414–19, 417.

120 Annette Runte, *Biographische Operationen. Diskurse der Transsexualität* (Munich: Wilhelm Fink, 1996), 197–8. An example is Victor Demole, ‘Transvestitisme démentiel androgyne’, *Revue médicale de la Suisse romande* 44 (1924), 25–32, confirming ‘the idea of an astonishing diversity of sexual psychic components determining transvestism’ (28).

121

Manfred Herzer , Magnus Hirschfeld und seine Zeit (Berlin/Boston: Walter de Gruyter, 2017), 202–9.

122 See Fischer, *op.cit.* (note 18), esp. ch. 5.

